# Acoustic camera system for measuring ultrasound communication in mice

**DOI:** 10.1016/j.isci.2022.104812

**Published:** 2022-07-21

**Authors:** Jumpei Matsumoto, Kouta Kanno, Masahiro Kato, Hiroshi Nishimaru, Tsuyoshi Setogawa, Choijiljav Chinzorig, Tomohiro Shibata, Hisao Nishijo

**Affiliations:** 1 Department of System Emotional Science, Faculty of Medicine, University of Toyama, Toyama 930-0194, Japan; 2 Research Center for Idling Brain Science, University of Toyama, Toyama 930-0194, Japan; 3 Laboratory of Neuroscience, Course of Psychology, Department of Humanities, Faculty of Law, Economics and the Humanities, Kagoshima University, Kagoshima 890-0065, Japan; 4 Katou Acoustics Consultant Office, Yokohama 225-0021, Japan; 5 Osawa Memorial Institute of Architectural Environmental Engineering, Kanto Gakuin University, Yokohama 236-8501, Japan; 6 Department of Human Intelligence Systems, Graduate School of Life Science and Systems Engineering, Kyushu Institute of Technology, Kitakyushu 808-0196, Japan

**Keywords:** Biological sciences, Techniques in neuroscience, Biological sciences research methodologies

## Abstract

To investigate biological mechanisms underlying social behaviors and their deficits, social communication via ultrasonic vocalizations (USVs) in mice has received considerable attention as a powerful experimental model. The advances in sound localization technology have facilitated the analysis of vocal interactions between multiple mice. However, existing sound localization systems are built around distributed-microphone arrays, which require a special recording arena and long processing time. Here, we report a novel acoustic camera system, USVCAM, which enables simpler and faster USV localization and assignment. The system comprises recently developed USV segmentation algorithms with a modification for overlapping vocalizations that results in high accuracy. Using USVCAM, we analyzed USV communications in a conventional home cage, and demonstrated novel vocal interactions in female ICR mice under a resident-intruder paradigm. The extended applicability and usability of USVCAM may facilitate future studies investigating typical and atypical vocal communication and social behaviors, as well as the underlying mechanisms.

## Highlights

• A new sound localization system for ultrasound vocalizations in mice was proposed
• Simpler recording setup and faster processing were achieved by utilizing phase lag
• Vocal interactions in a resident-intruder paradigm were analyzed with the system
• The system may facilitate future studies investigating social behaviors

## Graphical abstract

**Figure:**
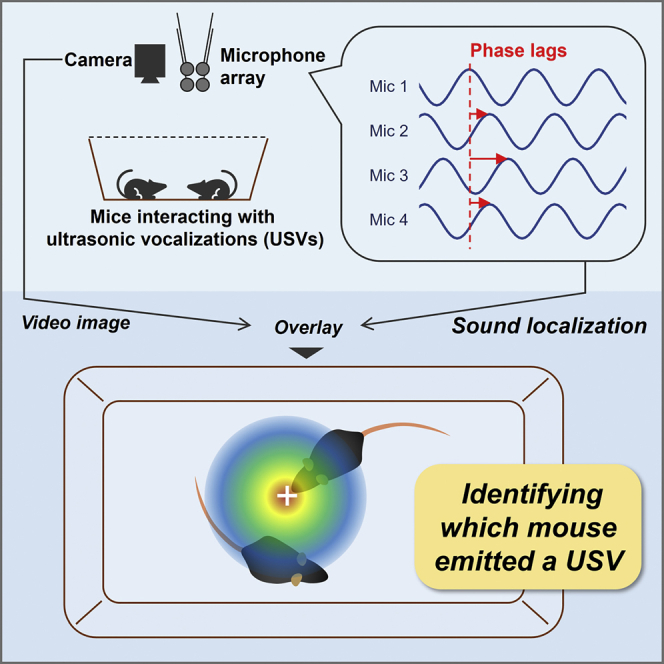

## Introduction

**Figure 1:**
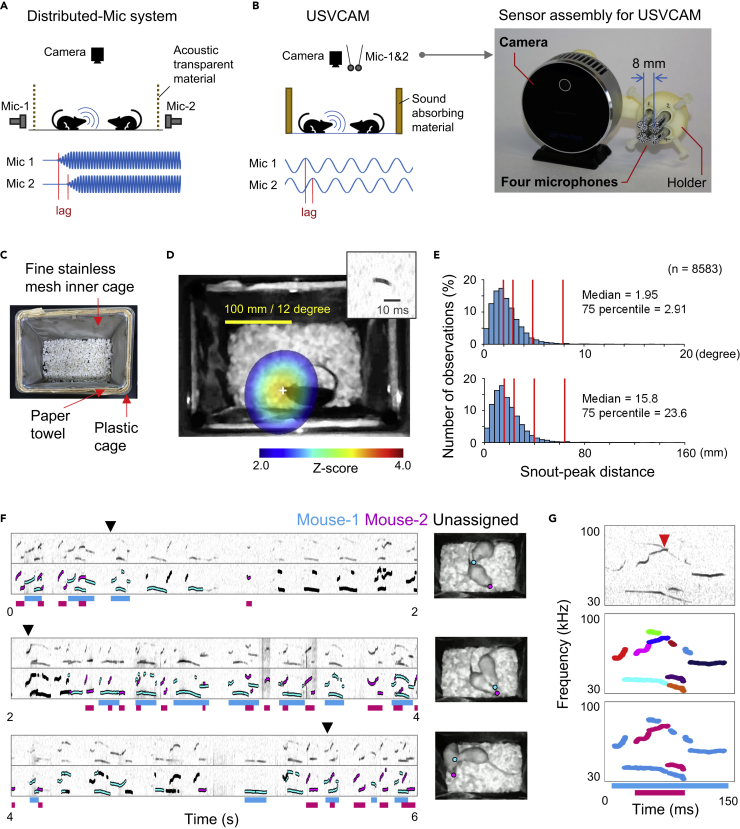
USV localization and assignment using USVCAM (A) A schematic showing the setup (top) and the signals received from the microphones (bottom) using the distributed-microphone (Mic) system. Only two microphones are shown for simplicity. Because the resolution of sound localization depends on the time lags of the sound arrival, microphones are located on the sides of the recording chamber, with acoustic transparent walls to maximize the lags. (B) A similar schematic (left) and a picture of the sensor assembly (right) of USVCAM. Because USVCAM utilizes phase lags of sound waves for sound localization, the microphone array can be set in one place. (C) A home cage equipped with the custom inner cage. (D) An example of sound localization of a USV segment (inset). The white cross signifies the peak of the spatial spectrum. (E) Distributions of the localization errors are shown in degrees (top) and millimeters (bottom). Red vertical lines indicate 50th, 75th, 95th, and 99th percentiles of the distributions, respectively. The error distributions separately calculated for B6 and ICR mice are shown in Figure S2. (F) An example of a USV assignment. The original spectrograms and those overlayed with the assignment results are shown. Bars under the spectrograms indicate syllables assigned to each mouse. The video frames at the black arrows are shown on the right. The snout positions are labeled by colored circles. (G) An example of the segmentation and assignment of the overlapping USVs emitted from different mice. Top, spectrogram; middle, the segmentation result (different colors indicate different segments); bottom, the assignment result. The frequency (y axis) ranges of all spectrograms in the figure D, F, and G are 30–100 kHz.

Ultrasonic vocalizations (USVs) are used for communication by many rodent species (Sales, 2010). Recently, USV communication in mice has received considerable attention as a powerful experimental model to investigate molecular, genetic, and neural mechanisms underlying social behaviors and deficits (Fischer and Hammerschmidt, 2011; Lahvis et al., 2011; Konopka and Roberts, 2016). Since differences in acoustic features of USVs are insufficient for discriminating individuals (Goffinet et al., 2021) and USVs are not associated with visually distinctive movements (e.g., opening mouth), it has not been feasible to identify which mouse in a group emits a certain USV. Therefore, USV communication has been left unexplored in most studies on social behavior in mice, despite its importance. Recent advances in sound localization technology in these studies have greatly facilitated the analysis of vocal interactions between multiple subjects (Neunuebel et al., 2015; Sangiamo et al., 2020). However, to date, sound localization systems for mouse USVs (Neunuebel et al., 2015; Heckman et al., 2017; Warren et al., 2018) are built around distributed-microphone arrays (Figure 1A; distributed-microphone systems), which require a special recording arena that is often equipped with reticulated (acoustic transparent) walls, surrounded by multiple microphones to investigate aspects of animal behavior. The distributed-microphone systems are also computationally demanding, practically requiring the use of a computer cluster for data processing (Warren et al., 2018). These technical requirements present a major obstacle for the application of such sound localization technology in established behavioral paradigms in many laboratories. To facilitate the application of sound localization technology in commonly used behavioral paradigms, we developed a novel system, named USVCAM (Figure 1B), which was inspired by the acoustic camera, a portable device that combines a camera and a compact microphone array to visualize sound sources on camera images. While the distributed-microphone system uses time lags of the sound arrival (Figure 1A, bottom) for the sound localizations, USVCAM can utilize the phase lags of sound waves (Figure 1B, bottom) thanks to a custom high-density microphone array (Figure 1B, right). The hardware design allowed the sensor assembly to be compact while maintaining the accuracy of sound localization. In addition to the recording simplicity, the processing speed in this system is considerably faster than that of the distributed-microphone system, since the computation time window is much smaller (single phase of sound wave Figure 1B). USVCAM also achieves more accurate localization by using a high-accuracy USV segmentation algorithm (Tachibana et al., 2020) recently developed by a team including the author K.K. to reduce noise and focus on the USV segments that require processing. Furthermore, the segmentation algorithm was modified to discriminate overlapping vocalizations from multiple subjects. Finally, we demonstrate the performance and effectiveness of USVCAM by analyzing USV communication in a home cage, which is challenging to perform with distributed-microphone systems.

## Results and Discussion

### Validation of USVCAM

Two strains of mice (C57BL/6 [B6] and ICR), which have distinct vocal communications and social behavior characteristics (Asaba et al., 2014; Golden et al., 2017), were used in the following experiments. First, to validate the performance of USV localization and assignment (i.e., to identify which animal vocalizes), we recorded the vocalizations from a single male mouse exploring an empty home cage of a female mouse (single mouse experiment), in which we were certain about the source of each USV. To achieve accurate sound localization, we reduced sound reflection by inserting disposable paper towels between a conventional plastic cage and a custom inner cage made of fine stainless mesh (Figures 1C and S1). Figure 1D shows an example of the spatial spectrum of a detected USV segment and the estimated source location (the peak of the spectrum) in a single mouse experiment (Videos S1 and S2; see Methods for details of the algorithm). The median errors of localizations were 1.95 degrees (direction from the microphone array) and 15.8 mm (Figures 1E and S2), which are comparable to those reported for previous systems (Neunuebel et al., 2015; Heckman et al., 2017; Warren et al., 2018). Furthermore, the localization process for each USV segment took approximately 0.1 s using a single consumer personal computer, which is more than 1000 times faster than the computation time (6 min/segment) reported in a previous distributed-microphone system (Warren et al., 2018), although direct comparison was not possible since these results were measured in different conditions including recording arenas.

Video S1. An example of USV localizations emitted from a male B6 mouse in a home cage of a female mouse, related to Figure 1

Video S2. An example of USV localizations emitted from a male ICR mouse in a home cage of a female mouse, related to Figure 1

In USVCAM, each USV segment is assigned to an individual whose average power at the snout position is significantly higher than that of other mice. We tested the assignment precision by performing simulations using data from the single mouse experiment with one to three virtual mice added at random locations within the cage (Table S1). Here, we defined “precision” as the percentage of segments correctly assigned to the real mouse (hit) in all assigned segments (hit + error). The simulation results indicated that the precisions were around 99%, which is consistent with the confidence threshold (0.99) used for assignment. We further confirmed that the precision was independent from the distance between mice, although the number of assigned segments became lower when the mice were close (Figure S3). We also counted the assigned segments during social interaction experiments between two or three real mice (Table S2). Despite the close interaction in the home cage, 25.7% to 86.7% (mean: 60.2%) of the segments were assigned, which is comparable with the performance of distributed-microphone systems (Neunuebel et al., 2015; Warren et al., 2018). The assignment ratios in ICR mice were relatively smaller than those in B6 mice because snout locations were sometimes unavailable when the snouts were hidden under another mouse during interactions between ICR mice; moreover, the ICR mice interacted closely more frequently (Table S3). Figure 1F shows an example of an assignment during the interaction between a pair of female ICR mice. Most USV segments were assigned to one of the mice, except for when the snouts of the mice were very close (see also Videos S3 and S4). To analyze the vocal patterns, the assigned segments were finally integrated into syllables based on the gaps between segments (bars under the spectrogram in Figures 1F and 1G). USVCAM separates segments around crossing points (red arrow head in Figure 1G; Figure S4), which helped discriminate overlapping USVs from different subjects (Figure S5). The ability to segment and localize overlapping USVs is another novel aspect of this system. In the following experiments, the maximum proportion of the overlapping syllables in a recording session was 28.4% (Table S4), underlining the significance of this novel function.

Video S3. An example of USV assignment during an interaction between a male (cyan) and a female (magenta) B6 mouse in a home cage, related to Figure 1

Video S4. An example of USV assignment during an interaction between two female ICR mice in a home cage, related to Figure 1

### Application of USVCAM

**Figure 2:**
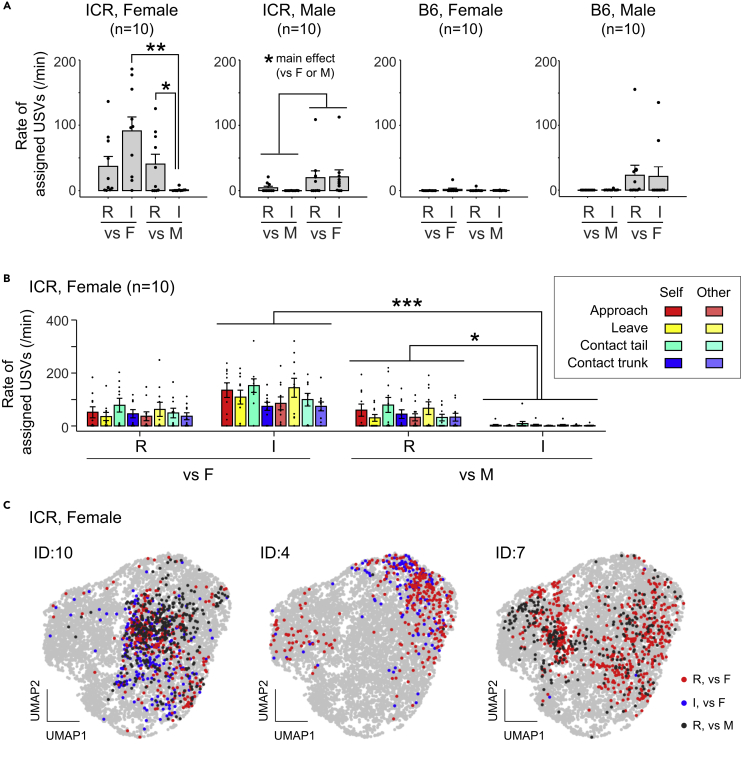
Analysis of mouse pair interactions under the resident-intruder (R-I) paradigm using USVCAM (A) Comparisons of rates of assigned USVs (number of assigned syllables per minute) of subjects in different social contexts. R, the subject was a resident; I, the subject was an intruder; vs F, the partner was a female; vs M, the partner was a male. Each dot represents an individual mouse. Error bars, standard error of the mean (s.e.m.); ∗∗*p* < 0.01, ∗*p* < 0.05, simple main effects analysis. (B) Rates of assigned USVs of female ICR mice during different actions by the subjects (self) and partners (other). See Figure S15 for the definition of the actions. Each dot represents an individual mouse. Error bars, s.e.m.; ∗∗∗*p* < 0.001, ∗*p* < 0.05, simple main effects analysis. (C) UMAP projection of the acoustic features of syllables extracted using the VAE (Goffinet et al., 2021). Examples of three ICR female mice are shown (ID, identity number of the mouse). Each point represents an assigned syllable. Red, blue, and black indicate the syllables of the subject recorded in different sessions. Gray points are all the other syllables recorded during the pair interaction experiments.

Finally, we evaluated the effectiveness of USVCAM in an actual behavioral experiment by using it to analyze USV communications under the resident-intruder (R-I) paradigm, which has been challenging with previous systems (Figure 2). All mice were tested with both a female (vs-F) and a male (vs-M) as both resident (R) and intruder (I). Figure 2A shows the mean rates of assigned USVs (number of assigned syllables/min) during each type of session (see Table S5 for statistical results). Interestingly, ICR females exhibited USVs even when they interacted with a female as an intruder and when they interacted with a male as a resident. However, it has previously been reported that in other strains the primary sender of vocalizations is the resident and the male during interactions between the same and different sex, respectively, in experiments using devocalization or anesthetization (White et al., 1998; Holy and Guo, 2005; Hammerschmidt et al., 2012). Furthermore, we compared the rates of assigned USVs during different actions of the self and other in the female ICR group, in which vocal interactions were most frequently observed (Figure 2B). Three-way ANOVA (action × vs F/M × R/I) revealed the significant main effect of the action (Table S6). The post hoc multiple comparison showed significantly more assigned USVs during own contact with other’s tail than the several other actions examined (Figure S6). The difference of the rate of USVs depending on the ongoing action is consistent with a previous report (Sangiamo et al., 2020). However, the ANOVA also showed significant main effect and interactions associated with the social contexts (vs F/M and R/I; Table S6), suggesting the rates of assigned USV was also modulated by the social context, even during the same types of actions (the result of the post hoc tests of the interaction between vs F/M and R/I was shown in Figure 2B). Finally, we analyzed acoustic features of USVs using dimension reduction by a variational autoencoder (unsupervised learning methods; Goffinet et al., 2021) to determine whether the distribution of acoustic features (i.e., the vocal repertoire) in ICR mice changes depending on social contexts (Figures 2C, S7, and S8). Results revealed no significant changes in the vocal repertoire depending on social contexts, although the vocal repertoire was dependent on individuals, as previously reported (e.g., Goffinet et al., 2021). We also tested different feature extraction methods to compare the vocal repertoire and obtained the similar results (Figures S9 and S10). To the best of our knowledge, this is the first report conducting a systemic analysis of complex acoustic features of syllables, including those that overlap (Table S4). Taken together, the results of USVCAM application revealed a novel characteristic of vocal communication in ICR mice under the resident-intruder paradigm.

There were several discrepancies between the results of the behavioral analysis in the present study and those of the previous studies. Female mice are known to exhibit USVs toward females (Maggio and Whitney, 1985), but in the present study, such female USVs were observed little in B6 (Figure 2A). Similar differences in the amount of USVs can also be found among recent previous studies using B6 female; females vocalized to females (F-F) more than males vocalized to females (M-F) in some study (Hammerschmidt et al., 2012), but the amount of such USVs was comparable between females and males in other study (Matsumoto and Okanoya, 2018). In another study, fewer amount of F-F USVs was observed than M-F USVs (Sasaki et al., 2020). Thus, the amount of vocalization seems to vary from study to study, and the causes are not well understood. The reason for the low vocalization of B6 females in this study is unclear. One possible explanation could be that, mice used as intruders are usually housed in group (e.g. Hammerschmidt et al., 2012; Matsumoto and Okanoya, 2018; Sasaki et al., 2020), but subject mice in the present study were deployed as both residents and intruders, and housed singly. Since the B6 females in this study exhibited low vocalization, we cannot rule out the possibility that B6 females vocalize toward males. Indeed, long-time recordings with sound localization revealed that B6 females vocalize when they encounter male mice (Neunuebel et al., 2015; Sangiamo et al., 2020). However, it is important to note here that resident individuals have been known as primary senders of USVs in short (3-min) recordings (Hammerschmidt et al., 2012) and that females have not been known to vocalize actively toward males in both short recordings (Maggio and Whitney, 1985) and relatively long (maximum 20-min) recordings (White et al., 1998). Therefore, active vocal interaction in ICR we found here is novel in any case. In particular, overlapping active vocal signals were observed in the ICR, and it was due to the advantages of USVCAM that they could be separated and localized.

In this study, we demonstrated the effectiveness of USVCAM in the home cage recording. The advantages of USVCAM will benefit the other various experiments. We also tested USVCAM in a different recording cage made of different materials and achieved similar sound localization accuracy (Figure S11). This application provides another example for reducing sound reflections for accurate sound localization with USVCAM. To record in the larger field (such as 50 × 50 cm) from the higher position, since the recorded vocalization sound will become smaller, better signal-to-noise ratio of the acoustic signal will be required. The signal-to-noise ratio could be improved by using more microphones or by replacing the microphones with more sensitive ones. The fast processing of USVCAM may also allow real-time USV localizations and assignments, with parallelization of the processes and use of a powerful computer. The real-time processing will be useful for new closed-loop experiments combining with feedback stimulations such as optogenetics, as well as the data compression for long-term recording (de Chaumont et al., 2019, 2021).

### Conclusion

In this study, we developed an acoustic camera system, USVCAM, which enables simple and fast USV localization and assignment by utilizing phase lags of sound wave. The system incorporates a recently developed USV segmentation algorithm with a modification that permits discrimination between overlapping vocalizations to achieve high accuracy. We applied USVCAM to analyze USV communications in home cages. Home cage recording is a popular method used in many experimental paradigms because it allows the observation of undisturbed behavioral expression and thus is ideal for investigating important aspects of social behavior (e.g., resident-intruder, sexual, and mother-infant interactions; Kikusui, 2013). However, previous systems have found this challenging using a conventional home cage. USVCAM revealed novel characteristics of vocal communication between ICR mice, suggesting that it will be effective in characterizing the social behaviors of various mice strains, such as those that have been genetically modified to establish disease models. USV is an important social signal in rodents (Sales, 2010), and previous studies have reported that USVs from different subjects (especially self and others) have different effects on brain activity and behavior (Rao et al., 2014; Matsumoto et al., 2016; Neunuebel et al., 2015; Sangiamo et al., 2020). Thus, sound source localization is fundamental for studying the dynamics of social behavior and its underlying mechanisms, as well as behavior phenotyping. Taken together, the extended applicability and usability of USVCAM may facilitate future studies investigating typical and atypical vocal communication, social behaviors, and the underlying molecular, genetic, and neural mechanisms.

### Limitations of the study

• The limitation of the present system for recording in the larger observation field is discussed above.
• At present, fully automated USV assignment was not possible, since a completely accurate snout location estimation of each individual mouse could not be achieved even with the state of the art of the deep-learning-based video tracking software. Manual curations of the video tracking results to correct errors such as identity switch were required in this study. Thus, further technical advancement in video tracking system may pave the way for real-time USV assignment with precision.

## STAR★Methods

### Key resources table

**Table:** 

REAGENT or RESOURCE	SOURCE	IDENTIFIER
**Deposited data**		

USVCAM sample data	This paper	https://doi.org/10.6084/m9.figshare.17121275

**Experimental models: Organisms/strains**		

Mouse: C57BL/6J	Japan SLC	C57BL/6JJmsSlc
Mouse: ICR	Japan SLC	Slc:ICR
Mouse: C57BL/6J	Jackson Laboratory Japan	B6J

**Software and algorithms**		

USVCAM software	This paper	https://doi.org/10.5281/zenodo.6626373
Python	Python Software Foundation	python.org; RRID: SCR_008394
MATLAB	The MathWorks, Inc.	mathworks.com; RRID: SCR_001622
AlphaTracker	Chen et al. (2020)	github.com/ZexinChen/AlphaTracker
Autoencoded vocal analysis	Goffinet et al., 2021	github.com/pearsonlab/autoencoded-vocal-analysis
R	R core team	r-project.org; RRID: SCR_001905

**Other**		

Plastic mouse cage	CLEA Japan	CL-0103-1
Ultrasound Microphone	ACO	TYPE 4158N
IR video camera	Intel	RealSense L515
Four-channel microphone amplifier	Katou Acoustics Consultant Office	BSA-CCPMA4-UT20
Analog-digital converter	National Instruments	PCIe-6374
Custom sensor holder	This paper	https://doi.org/10.5281/zenodo.6626373
Custom inner cage and cage lid	This paper	https://doi.org/10.5281/zenodo.6626373

### Resource availability

#### Lead contact

Further information and requests for resources and reagents should be directed to and will be fulfilled by the lead contact, Jumpei Matsumoto (jm@med.u-toyama.ac.jp).

#### Materials availability

This study did not generate new unique reagents.

### Experimental model and subject details

#### Animals

All animal experiments were performed with institutional ethics approvals (from the Institutional Animal Use Committee of Kagoshima University #L21007 and the Animal Experiment Committee of the University of Toyama #A2020MED-17). For the main experiment using home cages, we used 40 adult mice: 10 males and 10 females each of the C57BL/6J (B6) and ICR strains. Mice were purchased from Japan SLC (Shizuoka, Japan) at the age of 8 weeks and housed alone in a cage (182 × 260 × 128 mm, CLEA Japan, Tokyo, Japan) equipped with the custom inner cage (Figure 1C) for 1 week. Food (5L37 Rodent LabDiet EQ, PMI Nutrition International, MO, USA) and water were supplied *ad libitum*, and the animals were kept under a standard 12 h:12 h light-dark cycle. Soft paper chips were used for bedding (Japan SLC). Experiments were mainly conducted in the light phase, except for some of the recordings for system validation (the single mouse and three mice experiments, see below), which were conducted during the dark phase. The environment was maintained at a constant temperature (22–25°C) and humidity (50% ± 5%). For an additional experiment using a different type of cage (Figure S11), we used 3 adult B6 mice (one male and two females) purchased from Jaxon Laboratory Japan (Yokohama, Japan) at the age of 8 weeks. The male was housed alone in a cage (180 × 260 × 125 mm, NK system, Osaka, Japan) and the females were housed together in the same type of cage for 1 week before the recording experiment. The other rearing conditions was same as the main experiment. The main experiments and the additional experiment were conducted in Kagoshima University and University of Toyama, respectively.

### Method details

#### Recording

The sensor assembly (Figure 1B right) consisted of four ultrasound microphones (TYPE 4158N, ACO, Tokyo, Japan), a video camera (RealSense L515, Intel, CA, USA), and a custom three-dimensional (3D)-printed holder for the sensors. A square phased microphone array (8 mm on each side) was composed with the holder. The distance between the camera and the center of the microphone array was 54 × 9 × 6.5 (depth) mm (microphones were placed in front of the camera). The audio data was captured by each microphone, amplified with a four-channel microphone amplifier (BSA-CCPMA4-UT20, Katou Acoustics Consultant Office, Kanagawa, Japan), and sampled at 384 kHz using an analog-digital converter (PCIe-6374, National Instruments, TX, USA). An infrared image (resolution: 640 × 480 pixels; field of view: 70° × 55°) was captured with the camera at 30 Hz. The audio and video data were stored on the same PC (Elitedesk 800 G5 TW, Hewlett-Packard Inc., CA, USA) with video frame timestamps along the audio data for audio-video synchronization, using a custom recording software written in Python (Python Software Foundation, NC, USA).

Reducing sound reflection in recording environments is crucial for ensuring accurate sound source localization based on the time/phase lags (Figure 1B). To this end, recordings were performed in a soundproof box (63 × 53 × 84 [height] cm) with 20 mm thickness sound-absorbing melamine foam on the walls and ceiling (Figure S12). We also designed a custom inner cage for home cage recording (Figures 1D and S13). The inner cage was made from fine stainless mesh (mesh size: 150 mesh/inch). Clean disposable paper towels (Prowipe, Daio Paper Corp, Tokyo, Japan) were inserted into the space (5 to 10 mm) between the inner cage and the plastic home cage to suppress the influence of sound reflection (see Figure S1 for the effect of the inner cage). A custom cage lid with a clear mesh screen (Figure S13C) was used to prevent a mouse from escaping.

#### USV segmentation

For the segmentation (detection) of the USVs in the audio data, the USVSEG algorithm (Tachibana et al., 2020) was used with slight modifications. The USVSEG algorithm can robustly segment USVs from background noise by generating a stable spectrogram using the multitaper method and flattening the spectrogram by liftering in the cepstral domain. Although several different algorithms have been proposed for USV segmentation, a recent benchmarking study indicated that the USVSEG is comparable to the state-of-art method (Fonseca et al., 2021). We modified the USVSEG algorithm to separate crossing USVs emitted from different animals (Figures 1G and S4). First, a binary image of the spectrogram peaks was created (pixel size = 0.5 ms × 750 Hz) using the peaks obtained with the original USVSEG algorithm (Figure S4C). Second, the binary image was dilated twice and eroded once with a 3 × 3 square structuring element to connect the spatially neighboring components using imdilate() and imerode() functions in MATLAB (Mathworks, MA, USA; Figure S4D). Third, the corner points (i.e., the crossing points and edges) were detected using the corner() function in MATLAB (Figure S4E), and the pixels within the rectangles (3 × 15 pixels) centered on the corners were erased to cut segments at the crossing points. Finally, the boundaries of the subsegments were calculated by applying the watershed transform to the image using the watershed() function in MATLAB (Figure S4F), and the spectral peaks were grouped according to the boundaries (Figure S4G). Small segments with ≤3.0 ms were excluded from the subsequent analysis. We validated the algorithm with synthetic data, in which pairs of syllables recorded from a single mouse were overlapped (Figure S5). For test data generation, a pair of syllables recorded in a single mouse experiment were randomly selected, and the peaks of each syllable were detected. The peaks of the two sources were overlayed with a random time shift (± 20 ms). When the peaks from the different sources were close, one stronger peak was selected according to the USVSEG algorithm, and only one peak was selected within a narrow bandwidth. In total, 10,000 (5,000 from B6 and 5,000 from ICR mice data) overlapping syllables were generated, and the segmentation algorithms were applied. To test the effect of the modification of the segmentation algorithms, we compared the results between using the proposed algorithm and the algorithm without corner detection. To quantify the quality of the segmentation, the contamination ratio was defined as (1/*N*)×*∑n*_*i*_, where *n*_*i*_ and *N* represent the number of contaminated points (i.e., the points from the minor source) in the *i*-th segment and the total number of points, respectively.

#### USV localization

Using the conventional (Bartlett, or delay-and-sum) beamformer, the power (*P*) of sound arriving from a given spatial location (***r***) was calculated as follows (Krim and Viberg, 1996):

$$P\left(r,\;\omega ,t\right)={\left|w{\left(r,\;\omega \right)}^{H}x\left(\omega ,t\right)\right|}^{2}$$

$$x\left(\omega ,t\right)=\;{\left[X_{1}\left(\omega ,t\right),\;\ldots ,X_{m}\left(\omega ,t\right)\right]}^{T}$$

$$w\left(r,\;\omega \right)=\frac{a\left(r,\;\omega \right)}{\sqrt{a{\left(r,\;\omega \right)}^{H}a\left(r,\;\omega \right)}}\;$$

$$a\left(r,\;\omega \right)={\left[e^{-i\omega \tau_{1}\left(r\right)},\;\ldots ,e^{-i\omega \tau_{m}\left(r\right)}\;\right]}^{T}$$

$$\tau_{j}\left(r\right)=\;\left|r-r_{j}\right|/c$$

where *ω* and *t* are frequency and time indices, respectively. *X*_*j*_*(ω,t)* represents the short-term Fourier transform of the signal captured with the *j*-th (*j = 1,…, m*) microphone. ***r***_*j*_ is the position of the *j*-th microphone, *c* is the speed of sound, and *τ*_*j*_ is the expected time delay of the signal arriving at the *j*-th microphone from the sound source location ***r***. *T* and *H* denote the transposition and conjugate transposition, respectively. The beamformer shifts the signals captured by the microphones to compensate for the arrival time delays (i.e., the phase lags of the signals) using a steering vector ***a***, and sums the shifted signals to calculate the power (*P*). Thus, the function *P(****r****)* is expected to be the maximum when the location ***r*** overlaps with the actual sound source and is called the spatial spectrum of the sound. Because we are not interested in the absolute power of the signal but rather in the peak locations in the spatial spectrum for sound localization, the following normalized spatial spectrum *P*_*norm*_ was used:

$$P_{norm}\left(r,\;\omega ,t\right)={\left|\frac{w{\left(r,\;\omega \right)}^{H}x\left(\omega ,t\right)}{\left|x\left(\omega ,t\right)\right|}\right|}^{2}$$

The average spatial spectrum of a given segment *P*_*seg*_ (Figure 1D) was subsequently defined as follows:

$$P_{seg}\left(r\right)=\frac{1}{n}\overset{n}{\underset{k=1}{\sum }}P_{norm}\left(r,\;\omega_{k},t_{k}\right)$$

where *ω*_*k*_ and *t*_*k*_ represent frequency and time indices, respectively, of the *k*-th (*k=1, …, n*) peak of a USV segment in the spectrogram. To localize the USV segment, *P*_*seg*_ was calculated at each x-y location of the camera image. The depth (z) of the sound source locations were assumed to be constant (i.e., equal to the floor of the cage). To reduce computational load, the image was binned into 5 × 5-pixel bins, and *P*_*seg*_ were calculated for each bin. *P*_*seg*_ outside the image were also calculated for margins of 100 pixels on each side to correctly estimate the peaks of the spatial spectrum around the edge of the camera image. The resultant spatial spectrum often showed multiple peaks in the form of a square grid (Figure S14) owing to the periodicity of the sound wave and the square microphone array arrangement; this phenomenon is referred to as spatial aliasing. To avoid spatial aliasing affecting the result, we positioned the microphones as close as possible (Figure 1B) to maximize the distance between the multiple peaks in the spatial spectrum. Furthermore, we used a USV assignment algorithm that can deal with spatial aliasing (see below). The spatial spectrum was normalized with the z-score normalization to evaluate the saliency of the peaks. Peak locations (local maximums) in the spatial spectrum were searched as bins with equal values across the original spectrum and that filtered using the maximum filter (window size = 5 × 5 bins). A peak with a small height (z < 1.6, approximately < 95% in the cumulative distribution function of the standard normal distribution) was excluded from the sound source candidates. A USV segment without any clear peak (z < 2.3, approximately < 99% in the cumulative distribution function of the standard normal distribution) in the spatial spectrum was categorized as ‘unlocalized’ and was excluded from subsequent analysis. The snout-peak distance was defined as the distance from the snout of the mouse and the nearest peak in the spatial spectrum.

#### USV assignment

Figure S15 shows an overview of the algorithm for USV assignment for each USV segment. Initially, the snout-peak distance was calculated for each mouse, and the mice with snout-peak distances that were within the distance threshold (red dotted line) were selected as candidates of the sound source (Figure S15A). The distance threshold was set as 99th percentiles of the distribution of the snout-peak distances in the single mouse experiments (Figures 1E and S15A right). If the number of candidates was zero or one, the USV segment was categorized as ‘unassigned’ or assigned to the candidate mouse, respectively. If there was more than one candidate mouse, the following test was conducted among the candidates.

To assign the USV segment to one of the candidate mice following the screening procedure above, the average power at the snout of the candidates was first calculated (Figure S15B). The two highest powers among the candidates were compared using a two-tailed Wilcoxon signed-rank test. The resulting pvalue (*p*) was used for the assignment. In addition, we used the distance between the snouts of the best two mice (*d*) for the assignment because we found using a simulation that, with the same *p* value threshold, when the snouts of the two mice become closer, more assignment errors occurred (Figure S16). This may have been caused by an error in sound localization itself (Figure 1E) or an error in the video-based estimation of snout locations. Using the above two parameters *p* and *d* with the number of candidate mice (*N*_*c*_), the confidence for assigning the segment to the best mouse was calculated as the averaged precision of the assignment for similar conditions in the following simulation using the single mouse experiment data. In the simulation, *N*_*c*_-1 ‘virtual’ mice were assumed to be at random locations away from the real mouse at a distance *d*, and the precision (hit count/[hit + error counts]) of the assignment at the *p* value threshold *p* was estimated. The simulations were performed in advance for all possible combinations of *p*, *d*, and *N*_*c*_ (Figure S16), and the distribution of the precision was used as a lookup table for the confidence estimation to reduce the computational load. Since the lookup tables that had been separately calculated for B6 and ICR mice (Figure S17) were similar, we used a combined distribution (Figure S16) for assignment in this study. USV segments with a confidence level of >0.99 were assigned to the best mice, and the others were categorized as ‘unassigned.’

The confidence estimation may be inaccurate when the two best mice are located near two different peaks in the spatial spectrum because of spatial aliasing (Figures S14 and S18A). In such cases, although the absolute snout-snout distance (*d*) is large, both mice should be considered good candidates. To ameliorate the problem, we calculated the distance between snouts (*d’*) after converting the snout positions into their relative positions from the nearest peaks (i.e., ‘wrapping’ the positions in a period of the spatial spectrum; Figure S18B) and used *d’* for the confidence estimation.

#### Merging Segments into syllables

Rodent USVs consist of syllables, which have tens to hundreds of milliseconds durations with gap intervals (usually of > 30 ms). Previous studies categorizing syllable patterns reported that the patterns differed depending on the behavior, individual, and strain (Holy and Guo, 2005; Matsumoto and Okanoya, 2018; Goffinet et al., 2021). Thus, we merged the short segments (Figure 1G) into syllables after the assignment. Specifically, segments assigned to one mouse and ‘unassigned’ segments (if they existed) with gap intervals smaller than a given threshold (a minimum gap of 30 ms) were merged into a single syllable. The assignment rate was defined as the ratio of the time-frequency points assigned to the mouse to all the points in the syllable. We only used syllables with an assignment rate of 1.0 for the behavior analysis.

#### Audible broadband vocalization detection

Mice occasionally emit audible broadband vocalizations (BBVs; i.e., ‘squeaks’) during conspecific interactions (Finton et al., 2017). BBVs are loud broadband sounds characterized by a harmonic structure. To prevent misidentifying BBVs as USVs and misassigning USVs that significantly overlap with BBVs, we excluded the ultrasound segments that overlapped with the time intervals of BBVs from the analysis. BBVs were detected automatically using the following simple algorithm. First, the recorded sound was downsampled to 38.4 kHz. Second, the spectrogram (time window = 10 ms; frequency range = 2 to 16 kHz) of the sound was calculated. Third, continuous background noise and transient (impulse-like) broadband noise were reduced by subtracting the median value of each frequency bin and the median value of each time bin, respectively. Fourth, the spectrogram was filtered using a median filter (window size = 0.5 kHz) along the frequency axis. Finally, the maximum power in the spectrogram across the frequency was calculated for each time point, and the time intervals containing BBVs were estimated as the intervals in which the maximum power exceeded a certain threshold value (we used 28 dB in this study). To check the precision of the simple BBV detector, we selected three recording sessions that involved a relatively large number of BBVs (interactions between one pair of male ICR mice and two male-female ICR mouse pairs; see below for details of the recording experiment), and a blinded experimenter compared the results of the automatic detection with the manual annotations (Figure S19). A total of 161 BBVs were detected in the manual annotations. Of these, 160 overlapped with the automatic detection, and one was missed by the detector. The automatic detector had only 22 additional (false-positive) detections. Thus, the results confirmed that the simple detector can effectively exclude time intervals containing BBVs from the analysis. Table S7 shows the number of ultrasound segments that overlapped with the time intervals of BBVs in each of the social interaction experiments.

#### Calibrating microphone positions

For the above USV localization algorithm, microphone positions (***r***_*j*_) needed to be accurately calibrated. For the calibration, we searched the microphone positions that maximized the average power (*P*_*seg*_) at the snout locations for the 20 selected USV syllables in the single mouse experiment, as follows:

$$\underset{r_{1},\;\ldots ,\;r_{m}}{\text{argmax}}\overset{20}{\underset{l=1}{\sum }}P_{seg}\left(s_{l},\;l\right)$$

where *P*_*seg*_*(****s***_*l*_*,l)* represents the average power of the *l*-th selected syllable at the corresponding snout location (***s***_*l*_). The optimization was performed using the L-BFGS-B algorithm implemented in Scipy (Virtanen et al., 2020). The syllable that emitted different parts in the recording area was selected for appropriate calibration.

#### Video tracking

USV assignment requires frame-by-frame snout locations of each mouse. USVCAM users can choose any available high-precision video tracking software (such as DeepLabCut [Lauer et al., 2022], Social LEAP Estimates Animal Poses [SLEAP; Pereira et al., 2022], and Mouse Action Recognition System [MARS; Segalin et al., 2021]) to estimate snout locations. In this study, we used AlphaTracker (Chen et al., 2020) for tracking the locations of the snout and the other body parts. The software can relatively robustly track the locations of body parts of interacting mice using deep neural networks. We prepared labeled data to train the networks by manually annotating body parts (snout, tail-base, and left and right ears) and the bounding box of the mice in 1694 and 996 randomly selected frames from the videos of B6 and ICR mice, respectively. Different networks were trained for tracking B6 and ICR mice. The outputs of AlphaTracker were manually curated to correct occasional errors (e.g., switching mouse identities and flipping snout and tail-base locations) using custom software written in Python. The resultant trajectories of the snouts were filtered using a median filter (window size = 0.16 s) and used for USV assignment. The trajectories of the bounding box, tail-base, left and right ears, and snout were filtered using a locally estimated scatterplot smoothing (LOESS) filter (time window = 0.5 s) and used for behavioral event classifications (see below). Video S5 shows an example of the filtered trajectories used for behavioral event classification. These behavioral tracking data was synchronized with the audio data based on the video frame timestamps obtained with the custom recording software.

Video S5. An example of the video tracking result used for behavioral event classification, related to STAR Methods

#### Audible sound generation from recorded USVs

For intuitive data presentation, the audible sound was created according to the results of the USV segmentation and integrated with the videos (Video S1. An example of USV localizations emitted from a male B6 mouse in a home cage of a female mouse, related to Figure 1, Video S2. An example of USV localizations emitted from a male ICR mouse in a home cage of a female mouse, related to Figure 1, Video S3. An example of USV assignment during an interaction between a male (cyan) and a female (magenta) B6 mouse in a home cage, related to Figure 1, Video S4. An example of USV assignment during an interaction between two female ICR mice in a home cage, related to Figure 1) using a sound synthesis method proposed by Carruthers et al. (2013). First, the maximum peak of the USV segments at each time point was extracted. Then, the sound *x(t)* was generated as:

$$x\left(t\right)=a\left(t\right)\text{sin}\left[2\pi \int_{0}^{t}f\left(\tau \right)d\tau \right]$$

where *t* is time, and *a(t)* and *f(t)* are the amplitude and frequency of the peak, respectively. Thus, the generated sound contains no background noise. The frequency was linearly mapped from 0 to 192 kHz to 1 to 6 kHz to make the sound audible. The generated sounds were only used for visualization.

#### Experimental schedule

In the main experiments, after a 1 week habituation period, the social interactions between mice in the home cages were recorded. On the first day, each mouse was allowed to interact with another mouse of the same sex and strain (M-M and F-F contexts), both as resident and intruder (nine mice as residents and one mouse as an intruder were tested first). On the second day, each mouse was allowed to interact with another mouse of a different sex but the same strain (M-F and F-M contexts). In these cases, all individuals were used as both a resident and an intruder, and the order in which these roles were applied to the experiment was counterbalanced. For the recording, the home cage with a resident mouse was placed in the soundproof recording box, an intruder mouse was placed in the home cage, and the behaviors of the mice were recorded for 3 min. After the 2 days of paired social interaction recording, one mouse of each strain was recorded in a single mouse condition to obtain data for system validation and determine parameters for the USV assignment. In the single mouse experiment, a male mouse was placed in an empty home cage of a female mouse, and its vocalizations were recorded for 15 (B6) and 8 (ICR) min. We also tested the recording of a three-mouse interaction of each strain for 8 (B6) and 5 (ICR) min for system validation (Table S2).

In the additional experiment (Figure S11), fresh bedding from the female home cage was put in the recording cage. Then, the male mouse was placed in the recording cage and its vocalizations were recorded for 10 min.

### Quantification and statistical analysis

#### Data analysis

The number of syllables assigned completely (assignment rate = 1.0) was counted for each mouse for each recording. The rate of assigned USVs was calculated by dividing the syllable count by the recording duration. In addition, to check the relationship between USVs and specific actions, we defined the following five behavior events based on video tracking results: *approach*, *leave*, and *contact with the tail base*, *trunk*, and *snout* (Figure S20). Then, rates of assigned USVs during different behavior events were calculated separately. The rates of assigned USVs during contact with the snout were not analyzed because of difficulty in USV assignment when the snouts were very close. To quantify and compare the acoustic features (patterns) of syllables, we used a variational autoencoder (VAE), according to the method proposed by Goffinet et al. (2021). In this method, VAE learns to map single-syllable spectrogram images onto 32 latent features in an unsupervised manner. We reconstructed a spectrogram of an assigned syllable using the frequency and amplitude of each point in the segments of the syllable to enable the analysis of the acoustic feature of the syllables even when it temporally overlapped with the syllables emitted from the other mouse. In the method described in Goffinet et al. (2021), the spectrogram of a short syllable was stretched for encouraging the VAE to represent fine temporal details. We did not use the time stretch in the main analysis since the range of the syllable durations in the present dataset was not large. Instead we used fixed, relatively short time window (128 ms) to keep the original duration information. In total, 7960 single-syllable spectrogram images were reconstructed and used for VAE training and analysis. The distribution of the syllables in latent space was visualized using Uniform Manifold Approximation and Projection (UMAP) and the difference in the distributions (vocal repertoires) between a pair of different experimental conditions was quantified using maximum mean discrepancy (MMD), according to a previous study (Goffinet et al., 2021). Distributions with fewer than 10 syllables were excluded from the MMD analysis. In addition to the VAE described above, we also tested three other feature extraction methods: 1) the VAE with the time stretch, 2) features of the binned contour of a syllable, and 3) traditional acoustic features. In the first method, the same VAE was used with the spectrograms time stretched by a factor of $\sqrt{\frac{t_{max}}{t}}$, where *t* is the duration of the syllable and *t*_*max*_ was 128 ms. The time stretching encourages the VAE to represent fine temporal details (Goffinet et al., 2021). The second method was used for a similar unsupervised clustering in DeepSqueak (Coffey et al., 2019), one of popular software for USV segmentation. In this method, the contour of a syllable (the trace of the frequency of the maximum amplitude at each time point) was divided into 10 bins. Then, the frequency and shape (1st derivative) at each of the bins and the total duration of the syllable were used as the features. In the third methods, following traditional acoustic features of a syllable were used: median, minimum, and maximum frequencies, delta (max - min) frequency, standard deviation of the frequency, slope, sinuosity, mean amplitude, peak frequency (the frequency at the maximum amplitude) and duration (Coffey et al., 2019). The features of the second and third methods were z-scored for normalization. Statistical tests were performed using R (The R Foundation, IN, USA) and MATLAB. The significance threshold was set to 0.05. All of the statistical details can be found in the corresponding figure legends, figures, and tables.

## Data Availability

• Sample data recorded with USVCAM have been deposited at figshare and are publicly available as of the date of publication. DOIs are listed in the key resources table. The full datasets generated and/or analyzed for the current study are available from the lead contacts upon request. • All original code has been deposited at Zenodo and is publicly available as of the date of publication. DOIs are listed in the key resources table. • Any additional information required to reanalyze the data reported in this paper is available from the lead contacts upon request.
