# Supplementary material for: Acoustic camera system for measuring ultrasound communication in mice
Source: iScience. 2022 Jul 21;25(8):104812. doi: 10.1016/j.isci.2022.104812 (PMC9379670; doi:10.1016/j.isci.2022.104812)
Supplement: Document S1. Figures S1–S20 and Tables S1 — –S7 [file mmc1.pdf]

iScience, Volume *25*

Supplemental information

Acoustic camera system for measuring ultrasound communication in mice

Jumpei Matsumoto, Kouta Kanno, Masahiro Kato, Hiroshi Nishimaru, Tsuyoshi Setogawa, Choijiljav Chinzorig, Tomohiro Shibata, and Hisao Nishijo

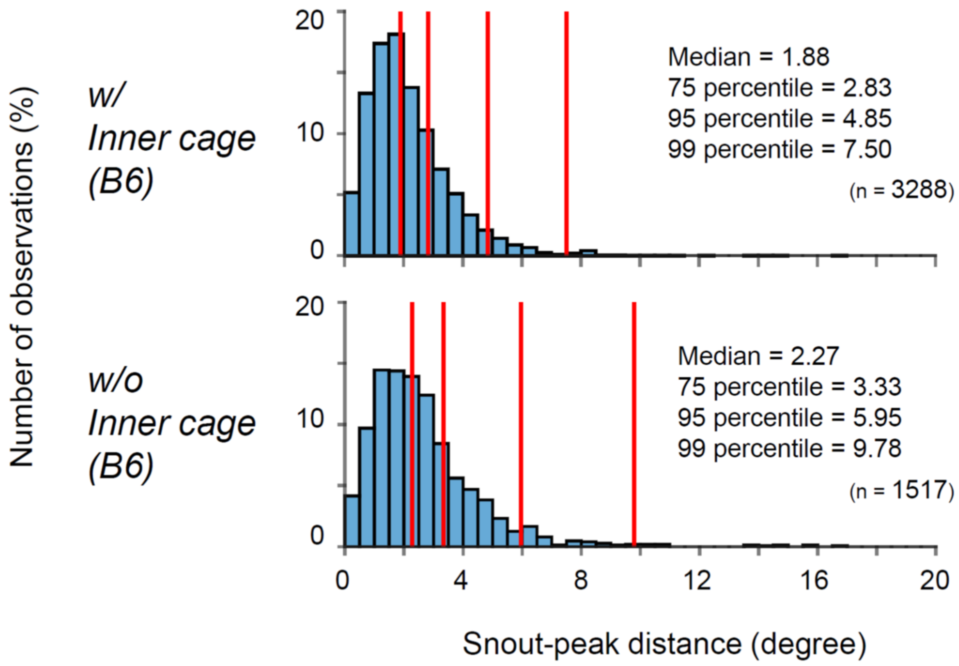

Figure S1. Error histograms similar to those in Figure 1E, in the home cage with (top) and without (bottom) the inner cage and paper towels in a B6 mouse, related to Figure 1.

Note that more outliers were observed without the inner cage (bottom), which demonstrates the effect of the inner cage.

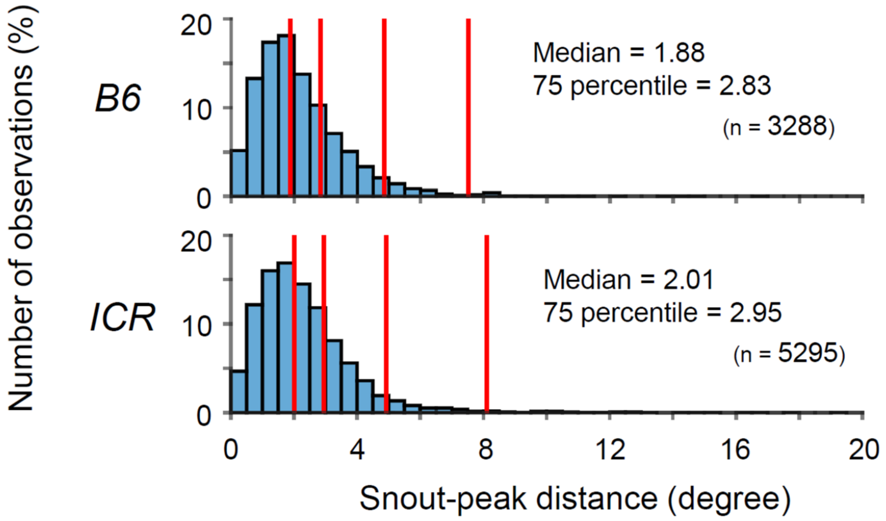

Figure S2. Error histograms similar to those in Figure 1E, calculated separately for B6 (top) and ICR (bottom) mice , related to Figure 1.

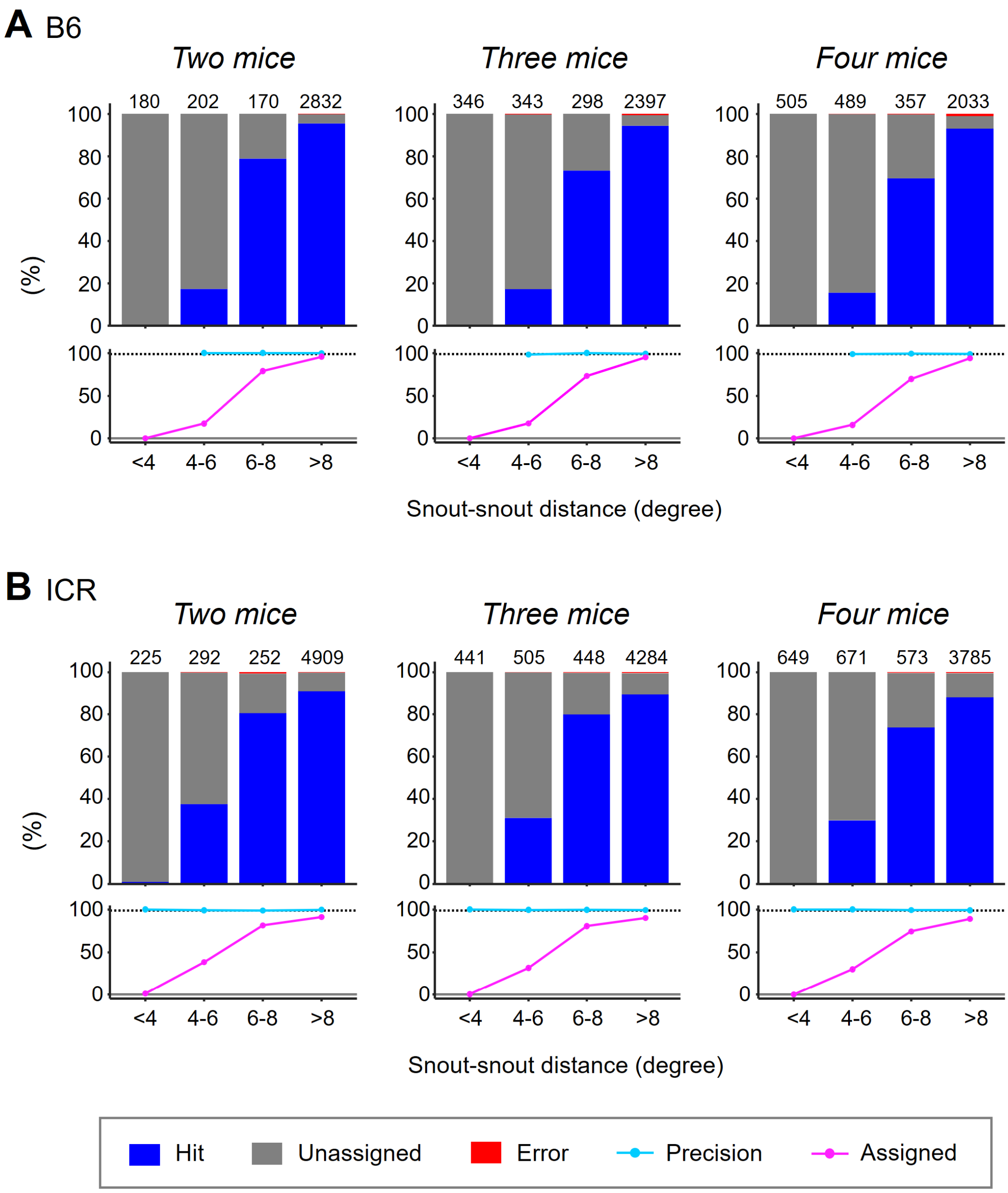

Figure S3. Relation between the USV assignment performance and the snout-snout distance in the simulations based on the single mouse experiment, related to Figure 1.

Each parameter in Table S1 was separately calculated for the distance between snouts of the best two mice with the highest average powers at their snout positions. Each column shows the data simulated with different number of mice in the cage. The numbers above the bars represent total number of USV segments tested in the corresponding conditions.

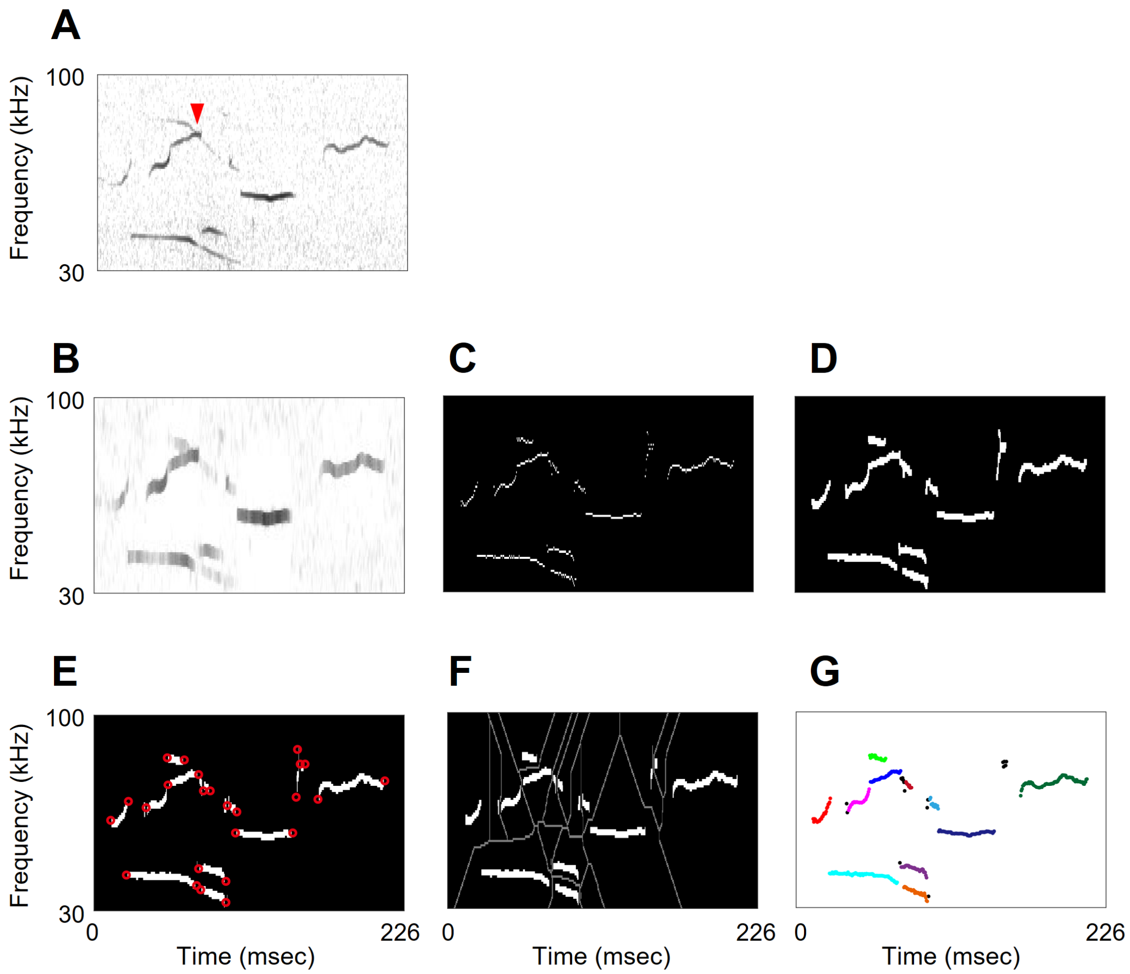

Figure S4. USV segmentation, related to Figure 1.

(A) A spectrogram of example USVs. Note that in this example, calls emitted from two different mice crossed at the red arrow. **(B–G)** The segmentation process. First, a filtered spectrogram was calculated **(B)**, and peaks in the spectrogram were extracted **(C)** using the USVSEG algorithm. Then, morphological dilation and erosion were repeatedly applied to connect the continuous peaks **(D)**. To separate the segments at the crossing points, corners of the images were detected and removed (**E**; red circles indicate the detected corners). Finally, the watershed algorithm was applied to find the boundary of the segments (**F**; gray curves indicate the boundaries of the segments). The resultant segments are shown in **G**. The colors represent different segments. Segments that were too short (≤ 3 ms; colored black) were excluded from subsequent localization processes.

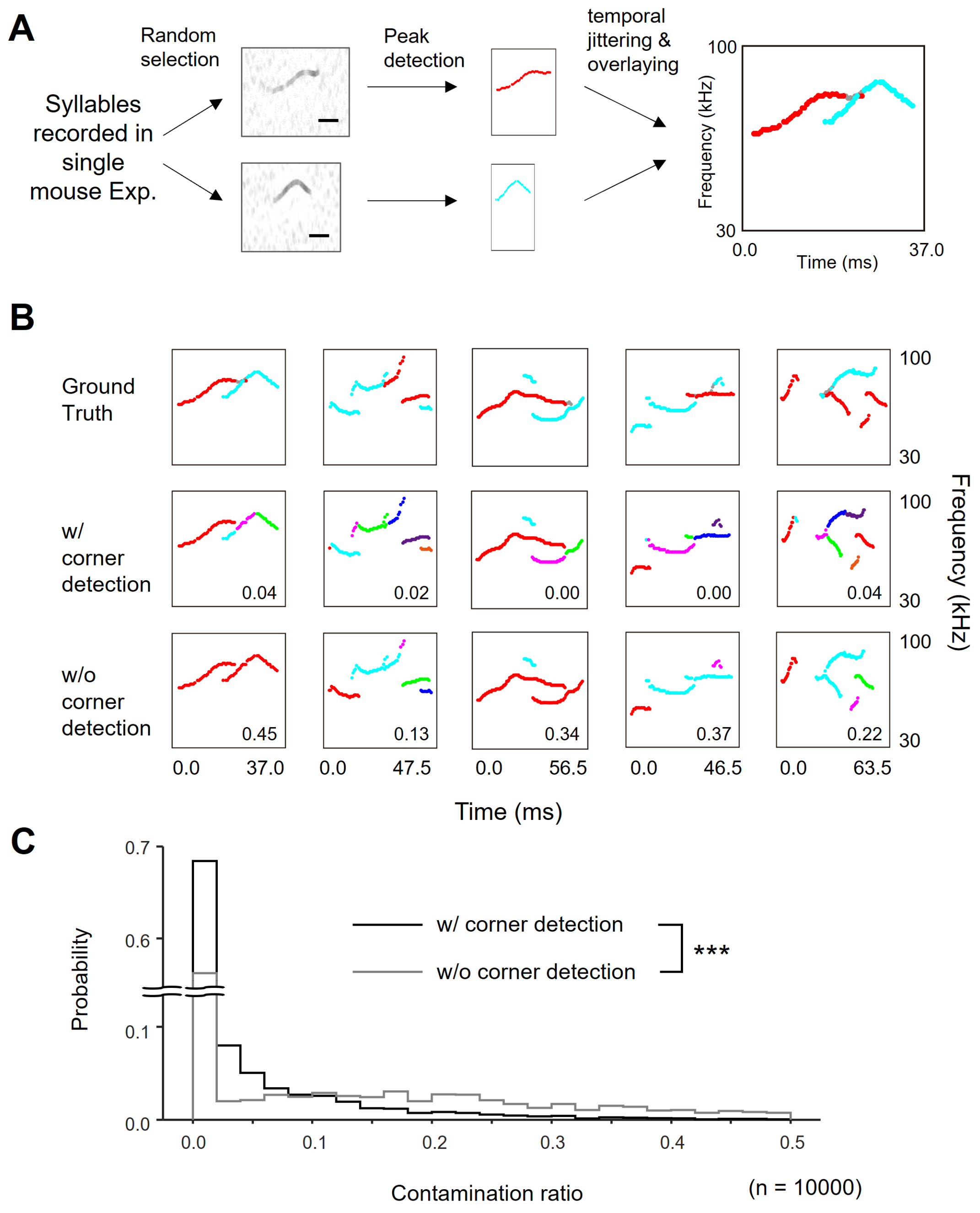

Figure S5. Effectiveness of the modified segmentation algorithm, related to Figure 1.
(**A**) Test data generation. Scale bars in the spectrograms = 20 ms. Gray peaks were excluded to simulate the peak search in the USVSEG algorithm. (**B**) Examples of the ground truth labels (top) and segmentation with (middle) and without (bottom) corner detection (Figure S1E) for separation at the crossing points. Different colors of the ground truth indicate different sources. Different colors in the segmentation results indicate different segments. The number in each segmentation result indicates the contamination ratio. (**C**) The distribution of contamination ratios in the segmentation with (black) and without (gray) corner detection. $^{***}p = 1.9 \times 10^{-117}$, two-tailed Wilcoxon rank-sum test.

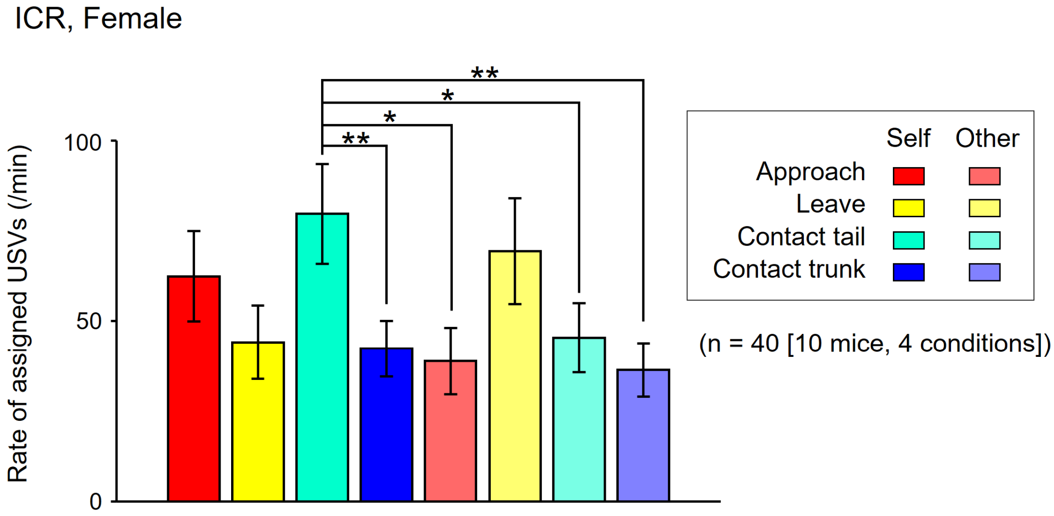

Figure S6. Comparison of the rates of assigned USVs among ongoing actions, related to Figure 2.

The same data in Figure 2B, but the different social contexts were combined. Error bars, s.e.m.; $**p < 0.01$, $*p < 0.05$, post hoc multiple comparison with Bonferroni correction.

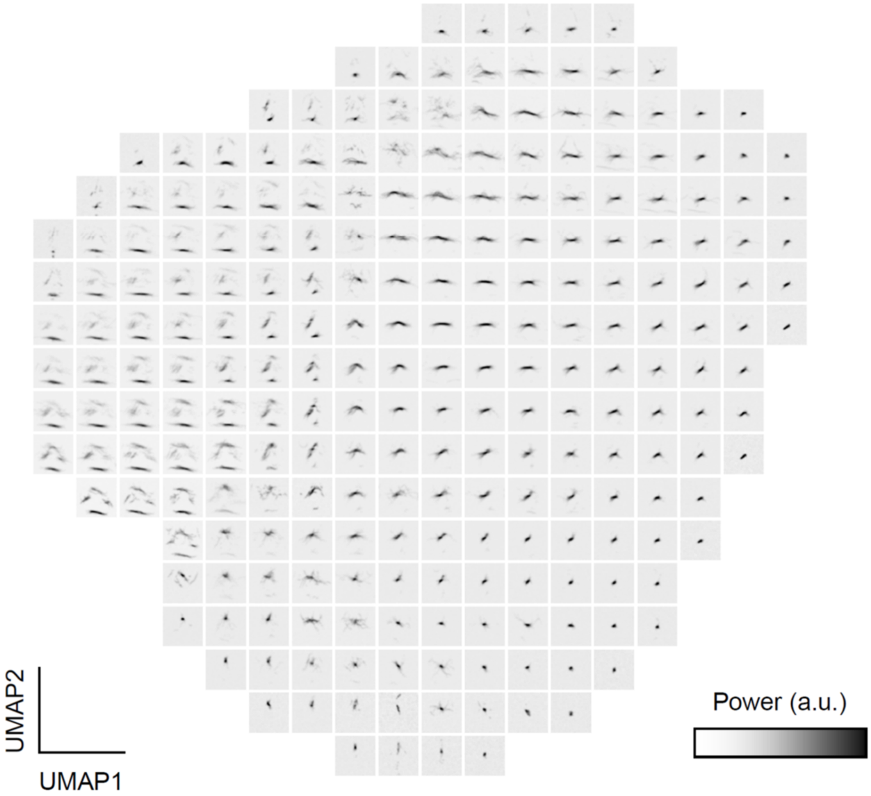

Figure S7. Mean spectrograms of the syllables for each location on the UMAP projection in Fig. 2C, related to Figure 2.

The frequency range of the spectrograms is 30 to 100 kHz, and the time range is 128 ms.

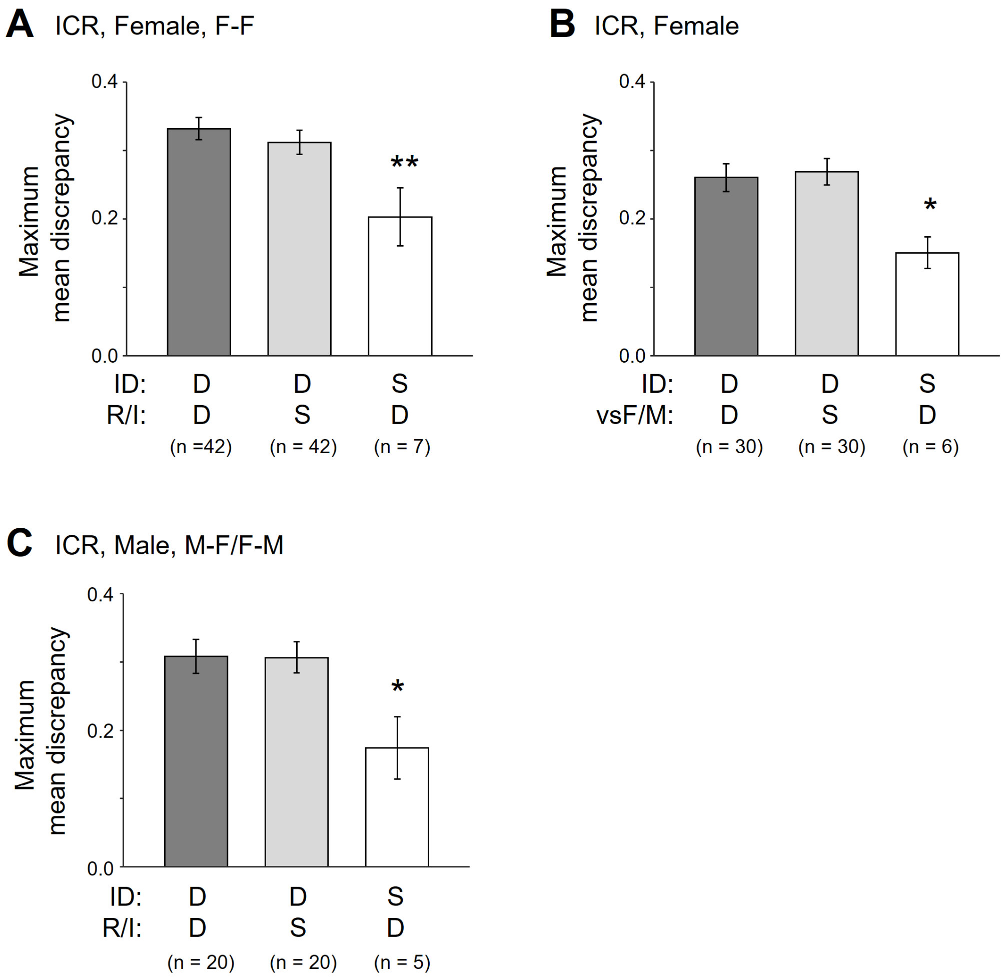

Figure S8. Quantitative analysis of the similarity of vocal repertoires within the same social contexts or individuals, related to Figure 2.

Dissimilarities in vocal repertoire calculated as maximum mean discrepancy (MMD) between pairs of distributions of the features of syllables (Fig. 2C) emitted by individuals. D, different; S, same. Error bars, s.e.m. (**A**) Comparisons of mean MMDs between groups of pairs of different IDs and different R/I labels (dark gray), pairs of different IDs and the same R/I labels (light gray), and pairs of the same IDs and different R/I labels (white) in female ICR mice in F-F sessions. $^{**}p < 0.01$, difference from pairs of different IDs and different R/I labels (dark gray) with Dunnett's test after one-way analysis of variance (ANOVA) ($F[2,88] = 4.154$; $p = 0.0189$). (**B**) Comparison of mean MMDs between groups of pairs of different IDs and different vsF/M labels (dark gray), pairs of different IDs and the same vsF/M labels (light gray), and pairs of the same IDs and different vsF/M labels (white) in female ICR mice in F-F, F-M, and M-F sessions. $^{*}p < 0.05$, difference from pairs of different IDs and different vsF/M labels (dark gray) with Dunnett's test after one-way ANOVA ($F[2,63] = 3.261$; $p = 0.0449$). (**C**) Similar comparison as (**A**) in male ICR mice in F-M and M-F sessions. $^{*}p<0.05$, difference from pairs of different IDs and different R/I labels (dark gray) with Dunnett's test after one-way ANOVA ($F[2,42] = 3.504$; $p = 0.0391$).

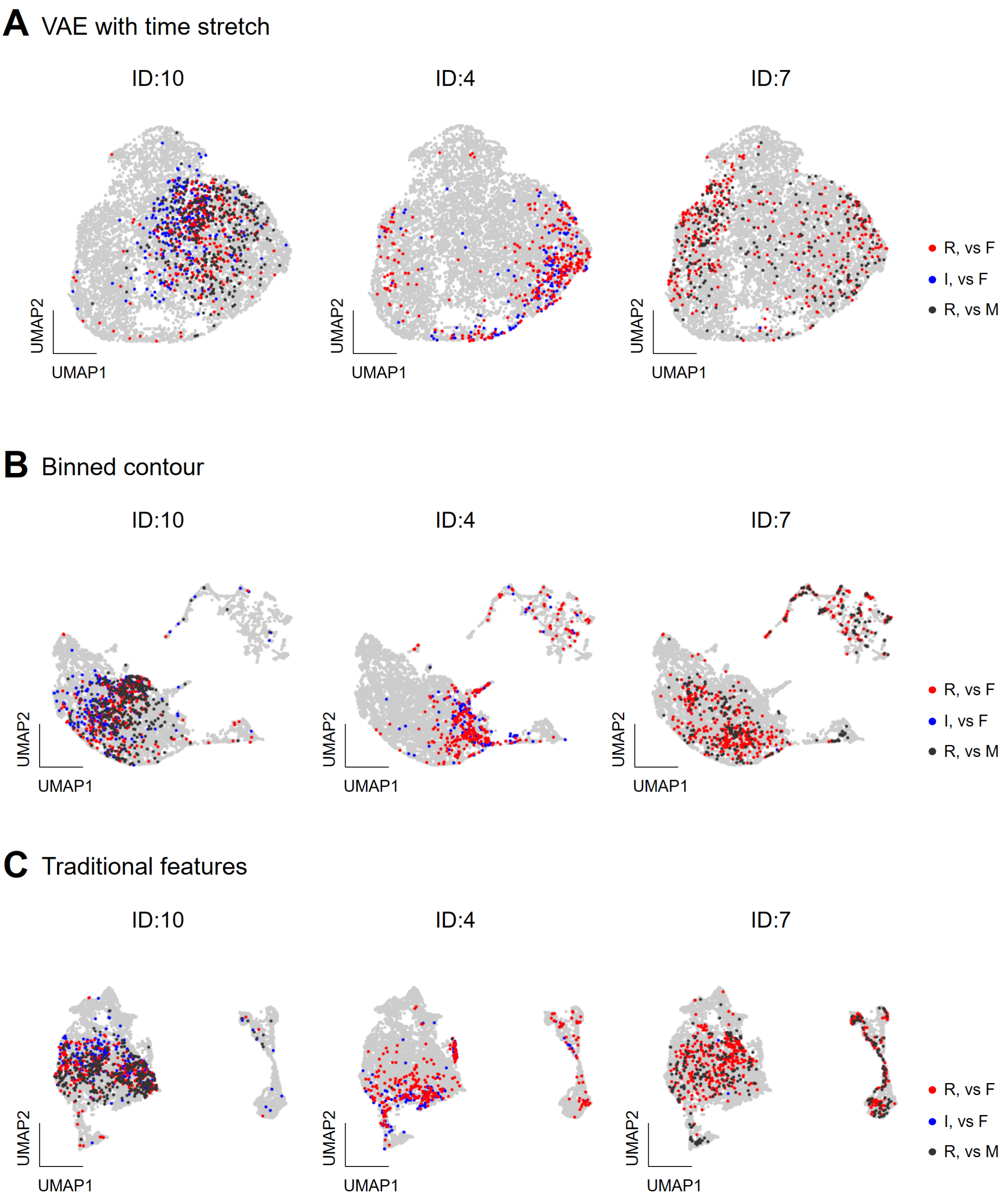

Figure S9. UMAP projections of the acoustic features calculated with different feature extraction methods, related to Figure 2.

See Methods for detail of the feature extraction methods. The other descriptions as for Fig. 2C.

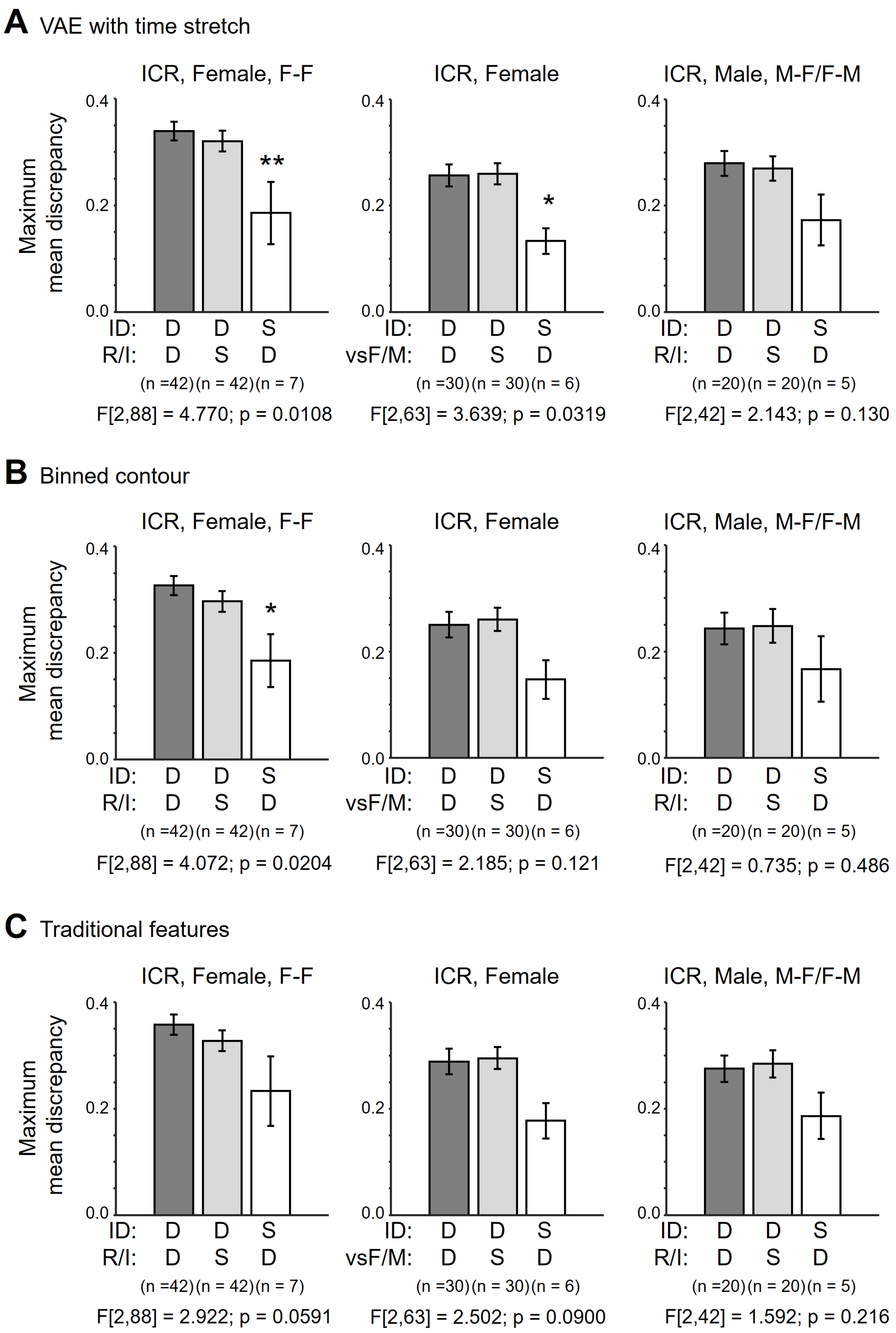

Figure S10. The same comparison of the vocal repertoires as Fig S8, using the different feature extraction methods, related to Figure 2.

See Methods for detail of the feature extraction methods. The result of the one-way ANOVA (F and p values) is shown below each graph. The other descriptions as for Fig S8.

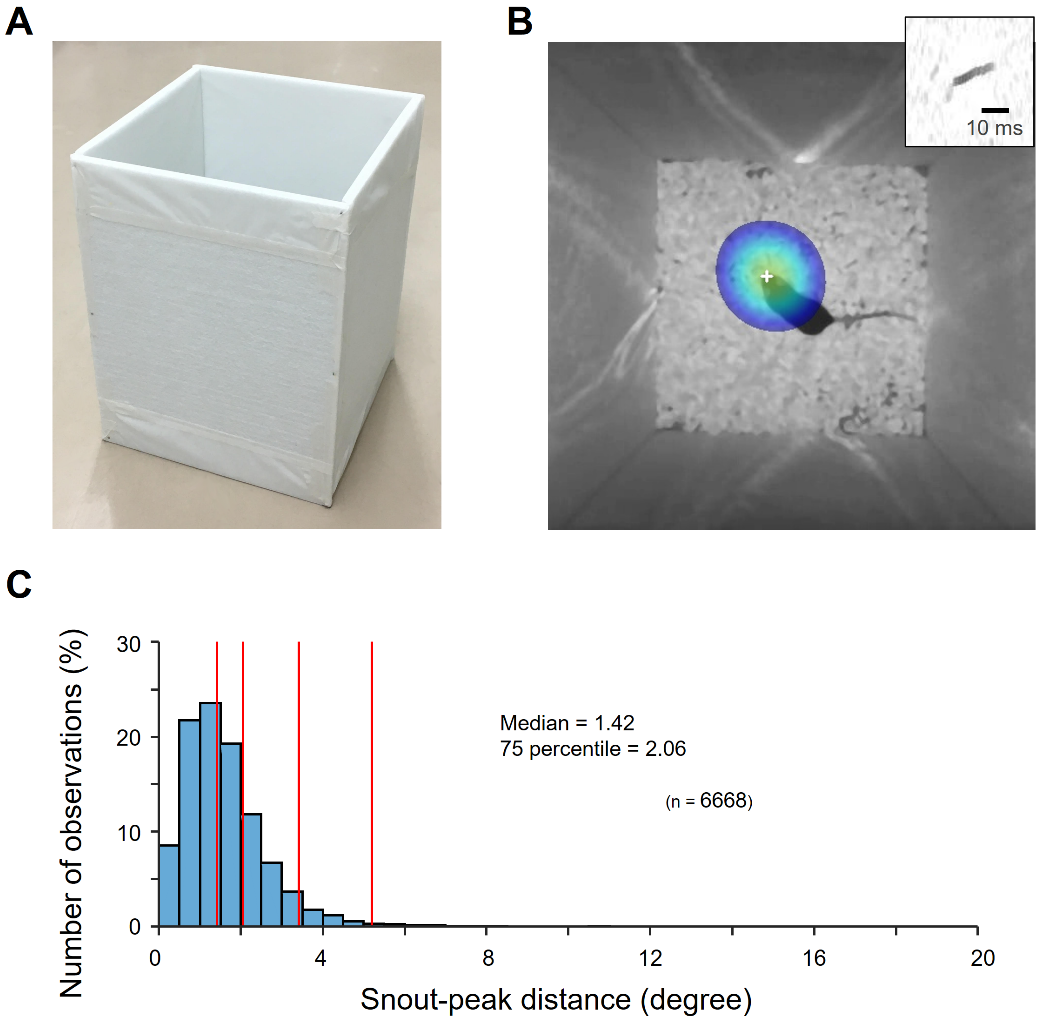

Figure S11. Performance of USVCAM in another type of cage, related to Figure 1.
(**A**) The recording cage (21 × 21 × 30 [H]) was made of acoustic felt panels (thickness: 10 mm). The inside was covered with the fine nylon mesh. (**B**) An example of sound localization of a USV segment (inset) in the cage. The white cross signifies the peak of the spatial spectrum. (**C**) Distributions of the localization errors in the cage. Red vertical lines indicate 50th, 75th, 95th, and 99th percentiles of the distributions, respectively. The USVs were recorded from a male C57BL/6J mouse.

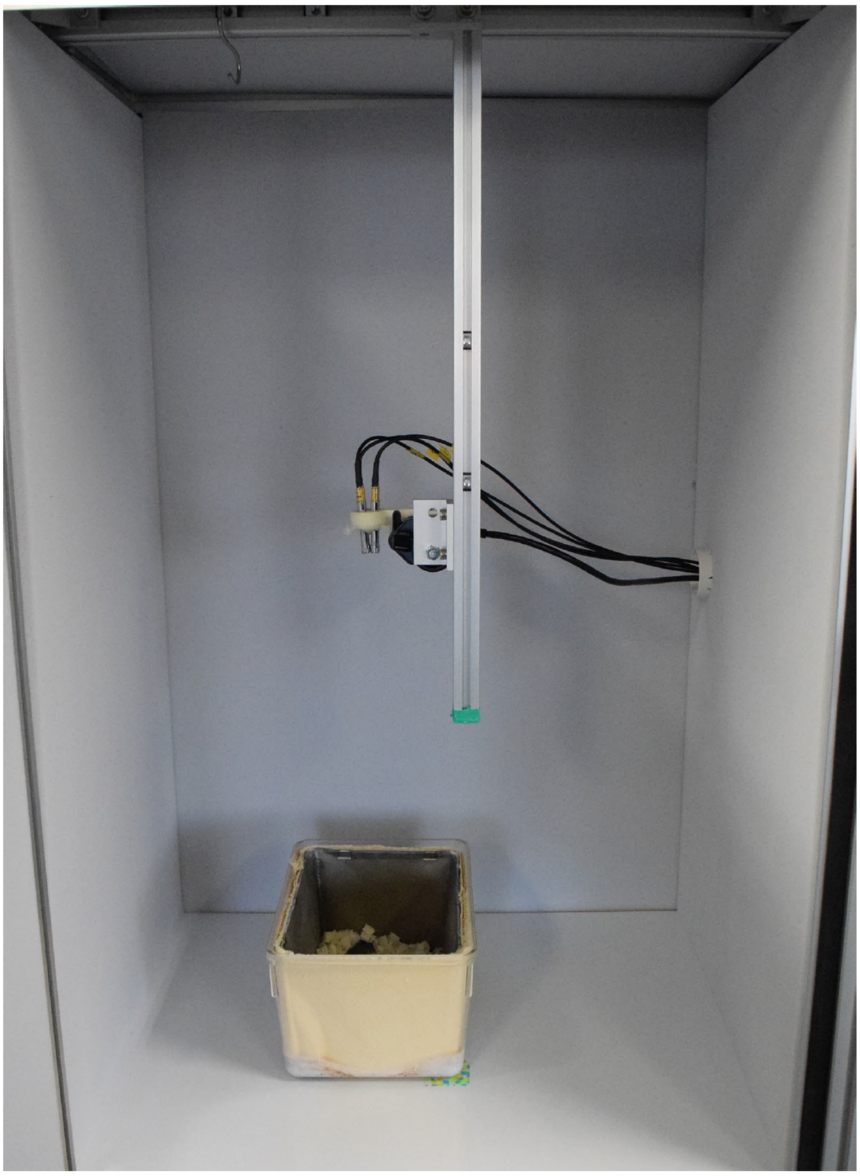

Figure S12. The soundproof box used in this study, related to STAR Methods.
The floor was made of polyvinyl chloride. The walls and ceiling were covered with sound-absorbing melamine foam to reduce sound reflections.

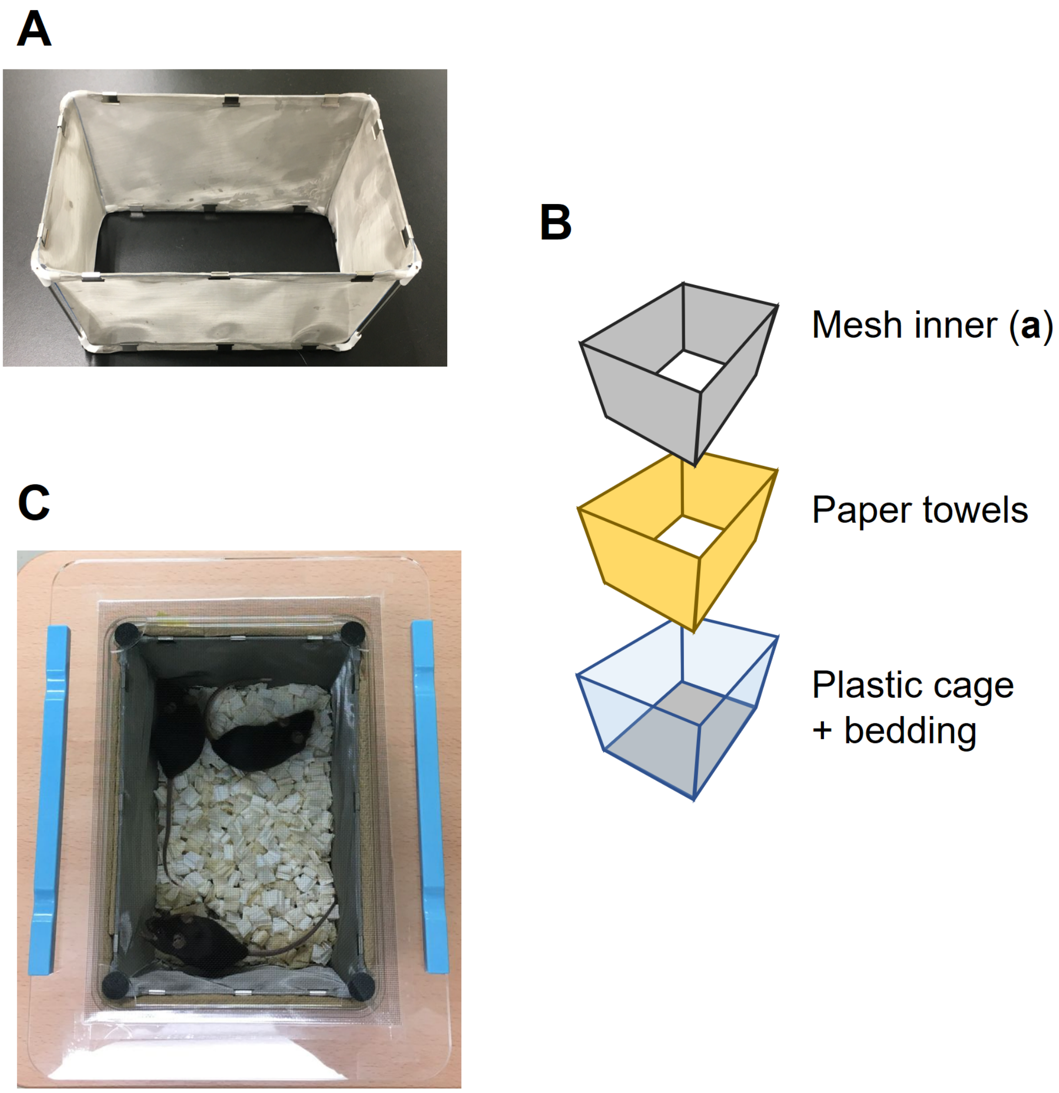

Figure S13. Details of the home cage recording, related to STAR Methods.
(**A**) The inner cage was made of fine stainless mesh. (**B**) An illustration of the layers of the cage. (**C**) The cage lid used for recording. A clear mesh screen was glued onto an acrylic frame. Rubber feet were attached to the corners of the lid to pin down the inner cage. The light blue bars are weights.

A

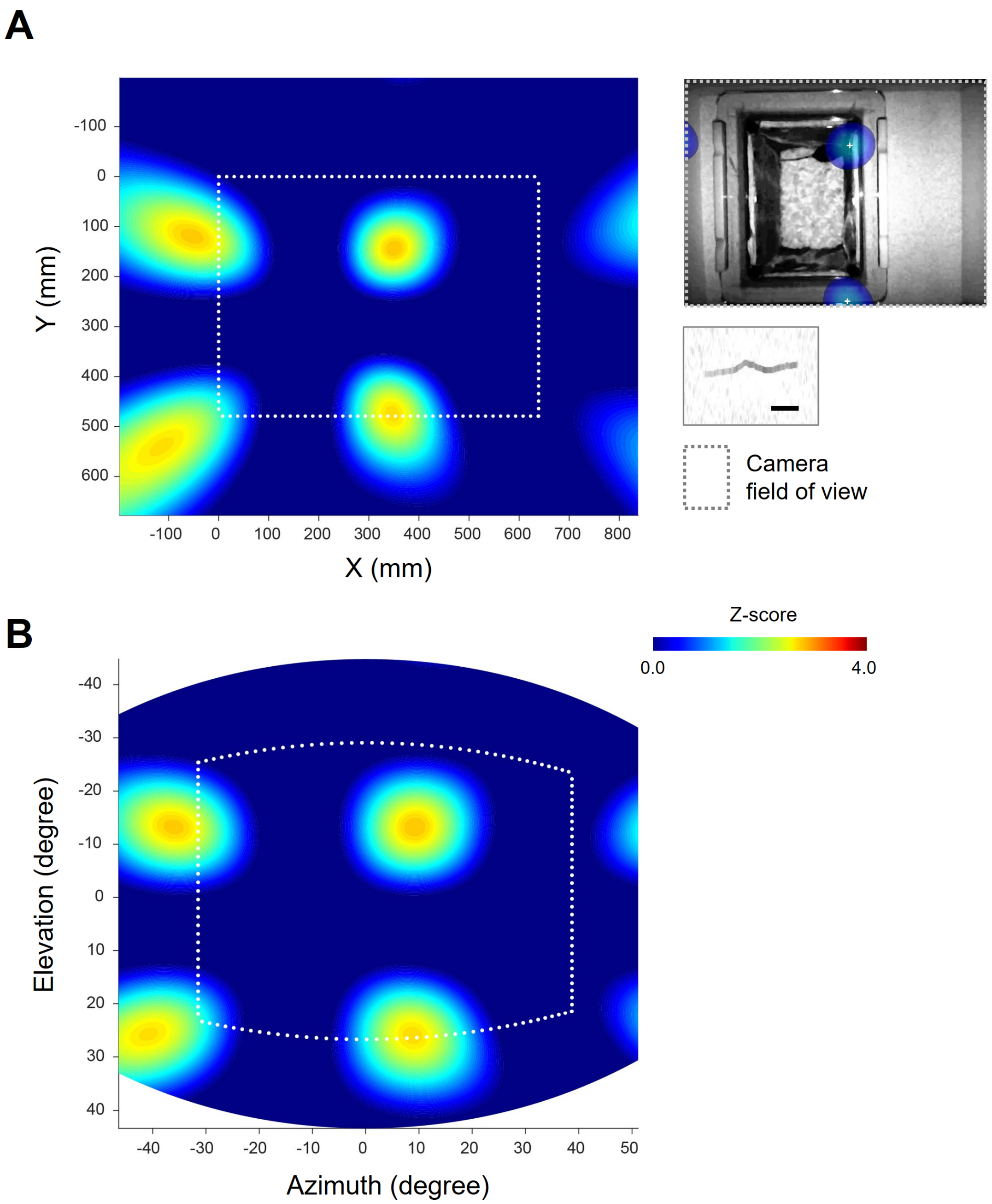

Figure S14. An example of the spatial spectrum shown in a wide field of view, related to STAR Methods.

(**A**) Spatial spectrum around the camera field of view. Inset shows the spatial spectrum overlayed on the video frame (top) and sound spectrogram (frequency range = 30 to 100 kHz, scale bar = 20 ms) of a USV segment. (**B**) The same spatial spectrum projected in the direction from the center of the microphone array. The peaks are located in a square grid.

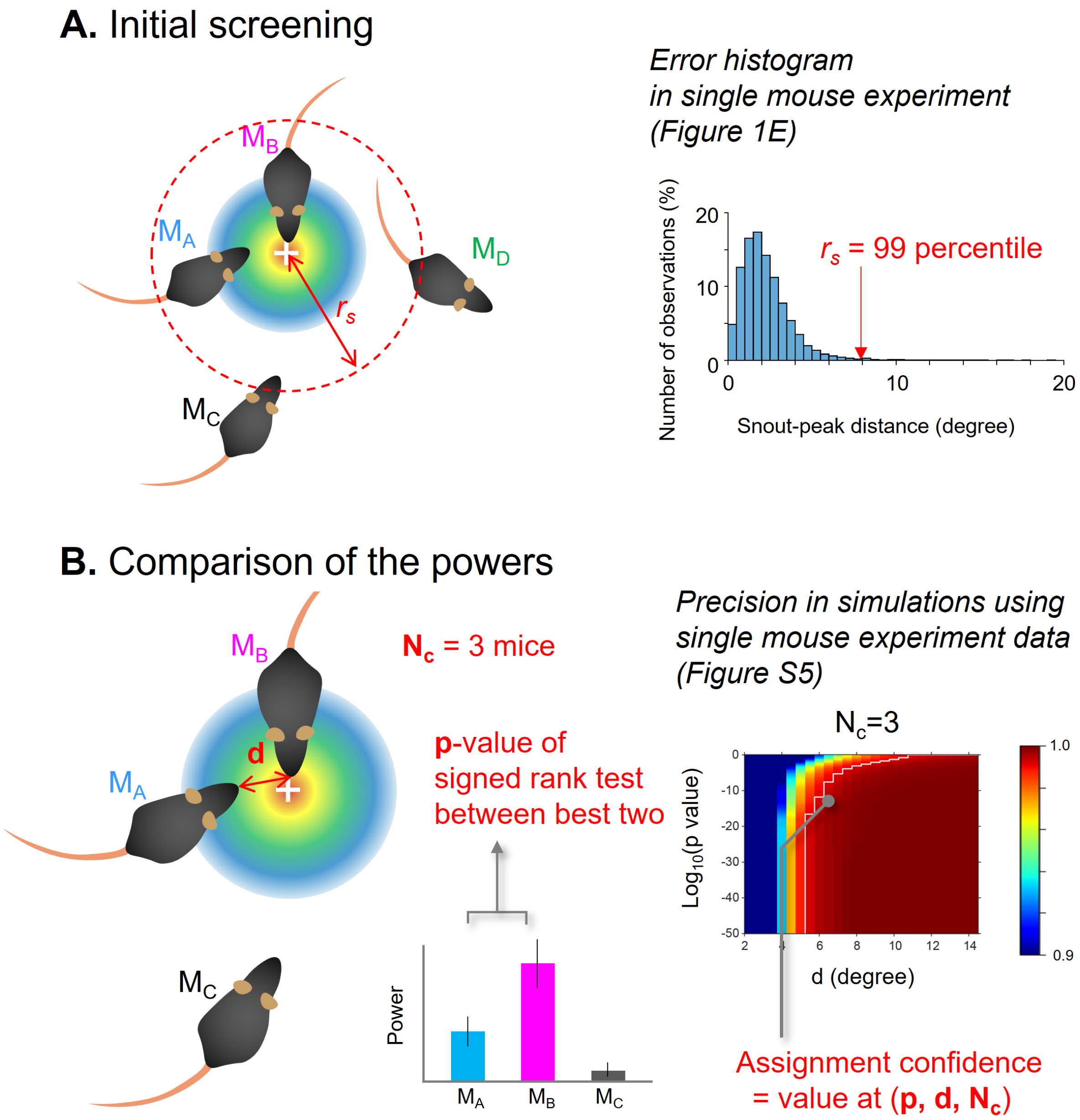

Figure S15. USV assignment, related to STAR Methods.

Assignment was performed in two steps: initial screening (**A**) and comparison of the sound powers at the snouts of mice (**B**). (**A**) First, mice with peak-snout distances that were longer than the threshold (r_s) were excluded from the candidates. $M_{A–D}$, IDs of mice. The white cross indicates the peak of the spatial spectrogram. (**B**) The USV segment was then assigned to the mouse with the highest power if the assignment confidence was higher than 99%. The confidence value was estimated using the *p*-value of the comparison between the best two mice (*p*), the distance between the snouts of the best two mice (*d*), and the number of candidate mice (N_c), based on simulations using the data of the single mouse experiments. See Methods for details.

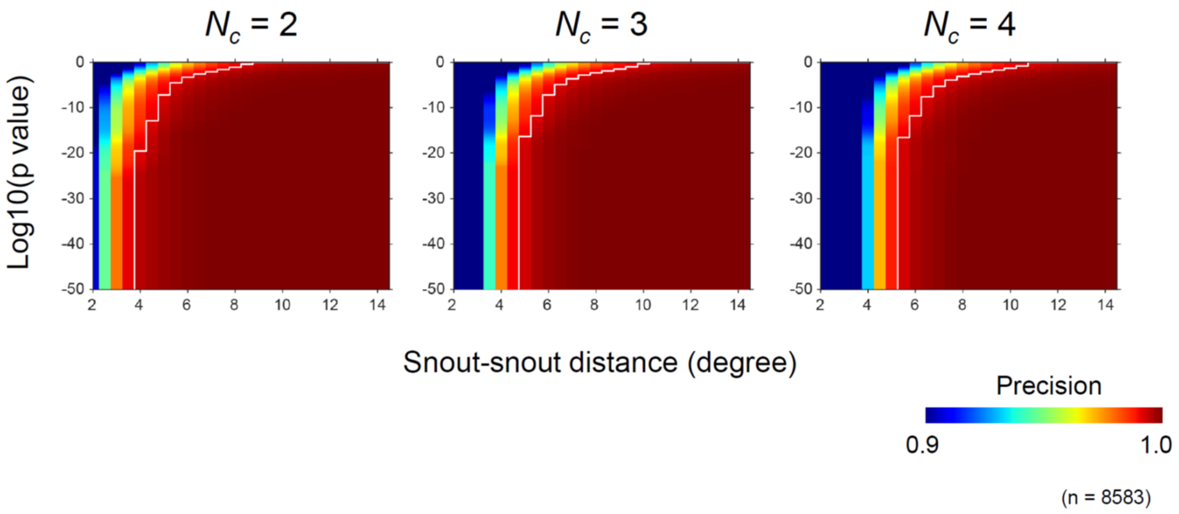

Figure S16. Distribution of assignment precision in the simulation using data obtained from the single mouse experiments, related to STAR Methods.

p, *p*-value of the sign-rank test comparing the sound powers at the snouts between the best two mice. N_c, the number of candidate mice. White curves indicate the border of 0.99.

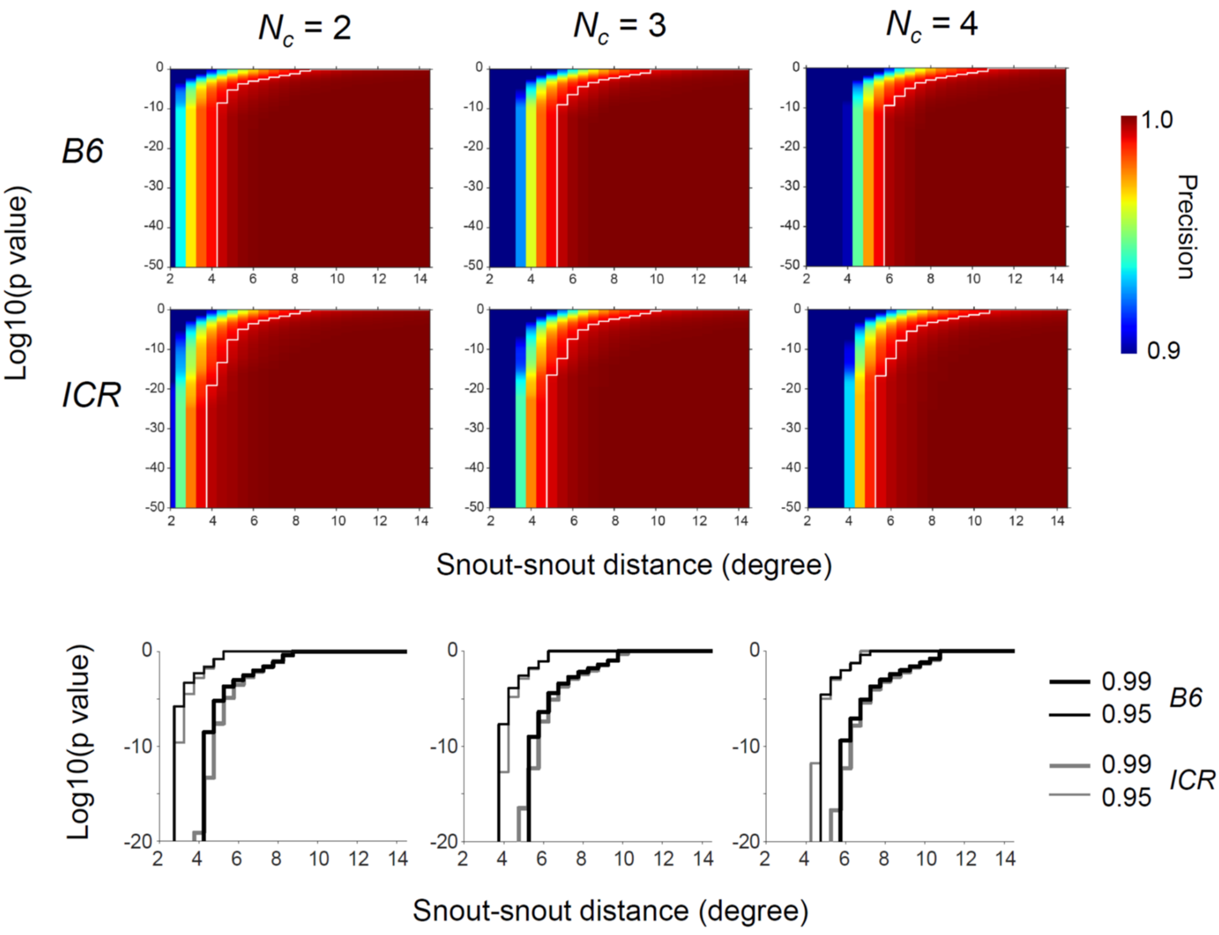

Figure S17. Distribution of assignment precision similar to that in Figure S16, calculated separately for B6 and ICR mice, related to STAR Methods.

Top: distribution of the precision. Bottom, comparison of the borders of 0.95 and 0.99 between strains. Because the simulation results for B6 and ICR mice were largely similar to those above, we used a combined distribution (Fig. S16) for the assignments in this study.

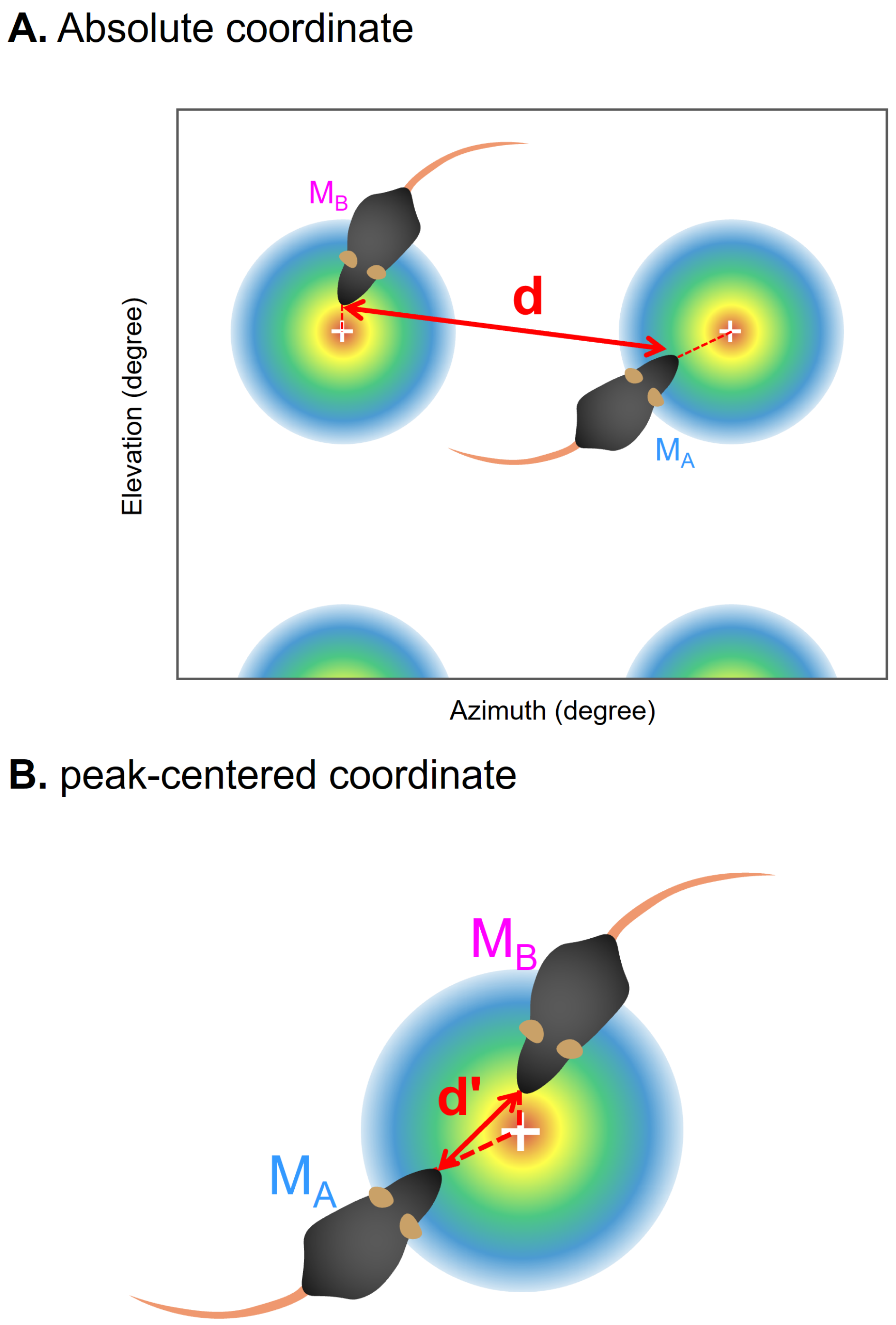

Figure S18. Spatial ‘wrapping’ for fixing snout-snout distance for the assignment, related to STAR Methods.

(**A**) An illustration of a case where mice are near different peaks. (**B**) The mouse locations in (**A**) are aligned to the peak-centered coordinates. The snout-snout distance in this space (*d’*) was used for estimating assignment confidence. M_A and M_B, IDs of mice.

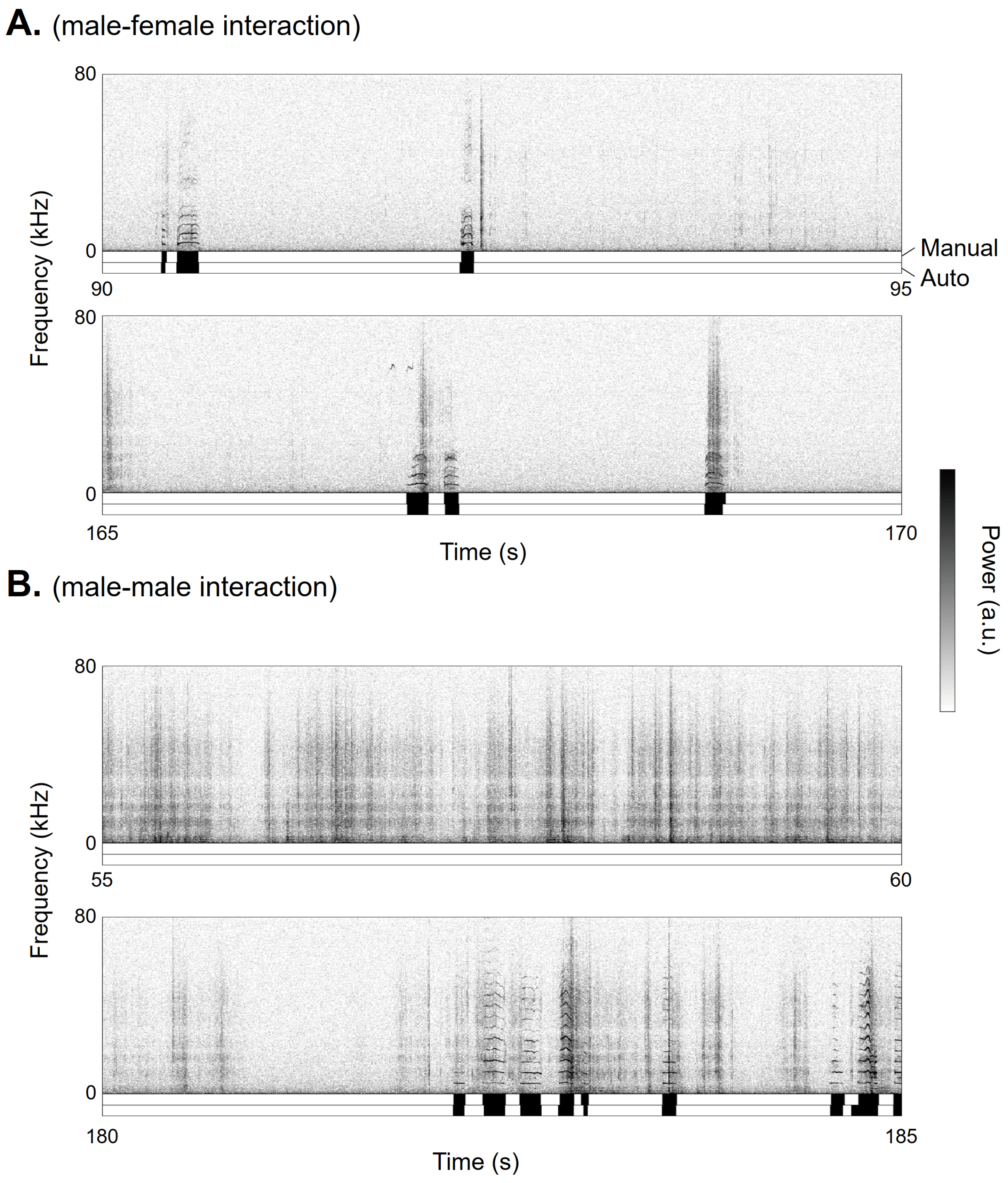

Figure S19. Examples of BBV detection using the automatic detector, related to STAR Methods. Examples of 5 s spectrograms are shown in male-female (**A**) and male-male (**B**) ICR mice interactions. Black bars in the first and second rows below the spectrogram indicate manual annotation and automatic detection of BBVs, respectively.

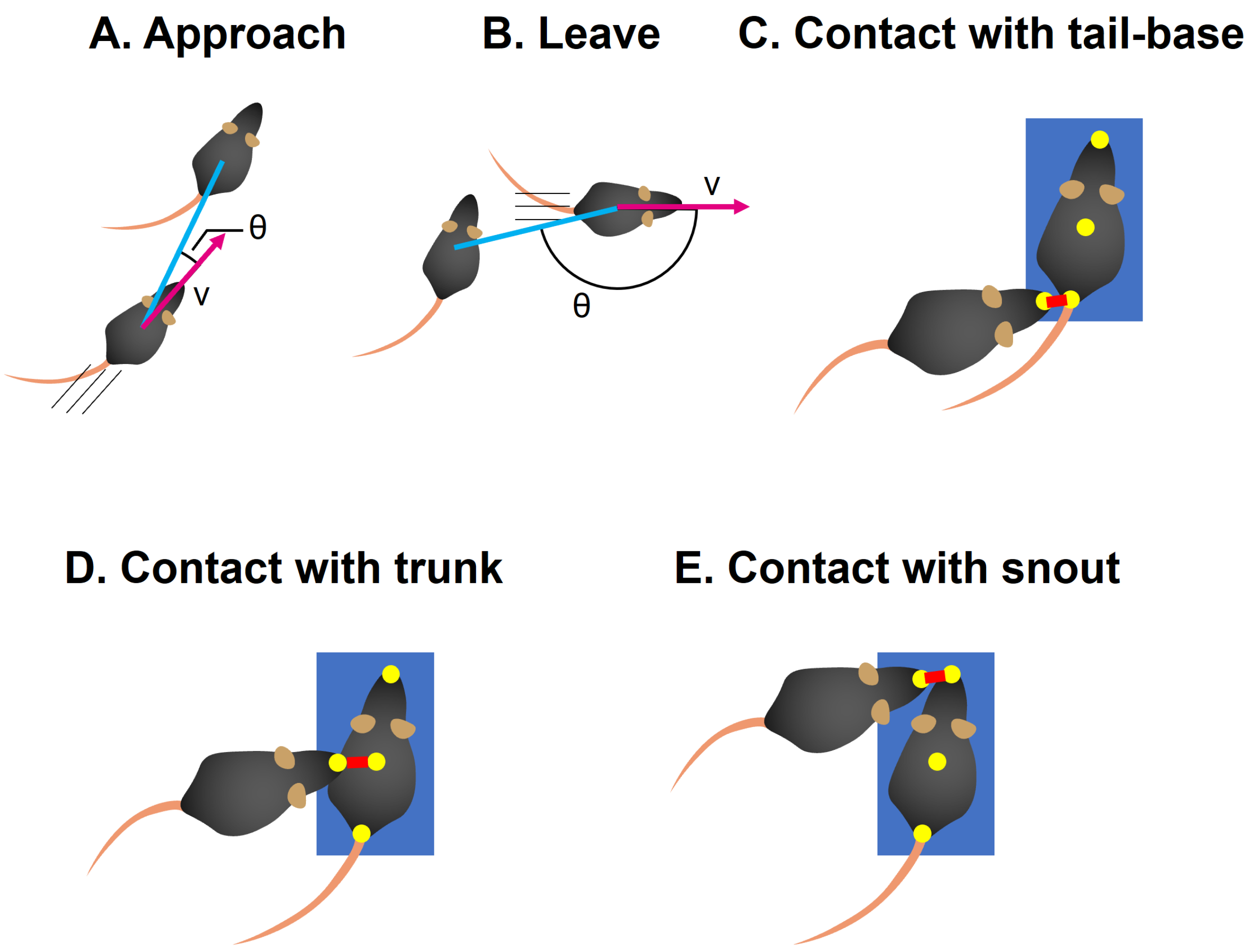

Figure S20. Definitions of the behavioral events, related to STAR Methods.
Approach (**A**) was counted when a subject's running speed (v) was > 50 mm/s, and the angle between the partner location and running direction (θ) was < 45 degrees. *Leave* (**B**) was counted when v was > 50 mm and θ was > 135 degrees. *Contact with the tail-base* (**C**), *trunk* (**D**), and *snout* (**E**) was counted when a subject's snout was inside the bounding box of the partner (blue shaded area), and the body part (yellow point) of the partner closest to subject's snout was the tail-base, the center of the bounding box, and the snout, respectively. The trunk position was calculated as the center of the bounding box.

Table S1. Assignment performance in the simulations based on the single mouse experiment, related to Figure 1.

			Assigned			Unassigned		
	Sub.	Total	Hit	Error	Precision	Low Conf.	Unlocalized	Pos. N/A
B6	2	3384	2873 (84.9%)	5 (0.1%)	**99.8%**	410 (12.1%)	65 (1.9%)	31 (0.9%)
	3	3384	2537 (75.0%)	15 (0.4%)	**99.4%**	736 (21.7%)	65 (1.9%)	31 (0.9%)
	4	3384	2213 (65.4%)	23 (0.7%)	**99.0%**	1052 (31.1%)	65 (1.9%)	31 (0.9%)
ICR	2	5678	4777 (84.1%)	17 (0.3%)	**99.6%**	501 (8.8%)	293 (5.2%)	90 (1.6%)
	3	5678	4347 (76.6%)	32 (0.6%)	**99.3%**	916 (16.1%)	293 (5.2%)	90 (1.6%)
	4	5678	3956 (69.7%)	31 (0.5%)	**99.2%**	1308 (23.0%)	293 (5.2%)	90 (1.6%)

Sub., number of subjects in the cage; one is the real mouse and the others are virtual mice randomly positioned in the cage.
Total, total number of USV segments detected.
Hit, number of segments correctly assigned to the real mouse.
Error, number of segments incorrectly assigned to one of the virtual mice.
Precision, percentage of Hit ÷ (Hit + Error).
Low Conf., number of segments in which assignment confidences were < 0.99.
Unlocalized, number of unlocalized segments.
Pos. N/A, number of segments emitted when the snout positions could not be estimated because of video-based tracking issues.
Audible call, number of segments that overlapped with audible calls.
Percentages in parentheses represent ratios relative to the total number of segments.

Table S2. Assignment performance in the home cage social interaction experiment, related to Figure 1.

				Unassigned		
	Sub.	Total	Assigned	Low Conf.	Unlocalized	Pos. N/A
B6	2 (F-F)	102	84 (82.4%)	14 (13.7%)	2 (2.0%)	2 (2.0%)
	2 (M-M)	30	26 (86.7%)	3 (10.0%)	0 (0.0%)	1 (3.3%)
	2 (F-M)	1795	1420 (79.1%)	339 (18.9%)	14 (0.8%)	22 (1.2%)
	2 (M-F)	2518	1823 (72.4%)	483 (19.2%)	29 (1.2%)	183 (7.3%)
	3 (M-FF)	970	578 (59.6%)	263 (27.1%)	52 (5.4%)	77 (7.9%)
ICR	2 (F-F)	16052	9813 (61.1%)	3203 (20.0%)	468 (2.9%)	2568 (16.0%)
	2 (M-M)	743	191 (25.7%)	288 (38.8%)	27 (3.6%)	237 (31.9%)
	2 (F-M)	8030	3881 (48.3%)	1760 (21.9%)	236 (2.9%)	2153 (26.8%)
	2 (M-F)	2897	1497 (51.7%)	759 (26.2%)	109 (3.8%)	532 (18.4%)
	3 (M-FF)	1607	569 (35.4%)	804 (50.0%)	69 (4.3%)	165 (10.3%)

Sub., number of subjects in the cage; the first and second letters in the parentheses indicate resident and intruder(s). M, one male; F, one female; FF, two females.

Total, total number of USV segments detected.

Assigned, number of segments assigned to one of the mice.

Low Conf., number of segments in which assignment confidences were < 0.99.

Unlocalized, number of unlocalized segments.

Pos. N/A, number of segments emitted when the snout positions could not be estimated because of video-based tracking issues.

Percentages in parentheses represent ratios relative to the total number of segments.

Table S3. Proportion of time spent in each behavioral event, related to Figure 2.

			Approach	Leave	Contact with tail-base	Contact with trunk	Contact with snout
B6	F-F	R	8.4 ± 3.3 %	9.1 ± 3.0 %	1.7 ± 1.6 %	3.0 ± 2.3 %	3.7 ± 1.7 %
		I	11.0 ± 3.6 %	6.8 ± 2.3 %	1.5 ± 1.7 %	1.5 ± 1.0 %	3.4 ± 1.7 %
	M-M	R	8.5 ± 2.4 %	9.7 ± 2.4 %	3.6 ± 1.8 %	3.4 ± 2.5 %	4.2 ± 2.2 %
		I	10.9 ± 3.1 %	8.9 ± 3.0 %	2.7 ± 2.7 %	1.9 ± 1.4 %	4.0 ± 2.2 %
	F-M	R	7.5 ± 4.1 %	6.8 ± 2.8 %	7.1 ± 4.4 %	5.6 ± 4.1 %	6.8 ± 3.3 %
		I	10.8 ± 4.6 %	9.1 ± 2.6 %	2.6 ± 2.2 %	2.1 ± 1.7 %	5.7 ± 2.5 %
	M-F	R	10.2 ± 5.9 %	5.9 ± 2.0 %	11.2 ± 8.5 %	5.6 ± 4.5 %	7.0 ± 4.8 %
		I	8.3 ± 3.0 %	10.3 ± 2.8 %	1.3 ± 1.3 %	2.5 ± 2.5 %	7.1 ± 4.7 %
ICR	F-F	R	11.9 ± 2.4 %	10.3 ± 3.7 %	10.5 ± 10.9 %	17.6 ± 14.0 %	10.3 ± 8.9 %
		I	11.9 ± 6.1 %	7.7 ± 2.3 %	13.4 ± 11.7 %	14.4 ± 14.4 %	9.5 ± 7.2 %
	M-M	R	10.3 ± 4.2 %	6.6 ± 2.3 %	5.6 ± 4.1 %	12.6 ± 6.6 %	19.5 ± 5.5 %
		I	6.6 ± 2.3 %	6.3 ± 1.7 %	2.6 ± 1.9 %	9.6 ± 5.5 %	19.1 ± 4.5 %
	F-M	R	14.9 ± 6.8 %	6.8 ± 2.5 %	17.8 ± 11.9 %	15.0 ± 5.6 %	19.2 ± 4.1 %
		I	8.0 ± 5.1 %	13.0 ± 5.6 %	7.0 ± 4.1 %	13.9 ± 9.0 %	18.0 ± 4.0 %
	M-F	R	12.9 ± 4.9 %	6.8 ± 2.1 %	15.2 ± 10.2 %	15.3 ± 7.3 %	21.1 ± 6.5 %
		I	7.1 ± 2.4 %	8.7 ± 3.0 %	3.8 ± 2.4 %	9.9 ± 3.5 %	23.5 ± 6.9 %

First, second, third column represent the strain, the experimental condition (the first and second letters indicate resident and intruder; F, female; M, male), and if the subjects were resident (R) or intruder (I), respectively. See Figure S15 and Methods for the definitions of the behavioral events. The data represented as mean ± SEM. The number of recording sessions was 10 for each experimental condition in each strain.

Table S4. Proportion of overlapping USVs, related to Figure 2.

		N	Mean	Min	Max
B6	F-F	0	-	-	-
	M-M	0	-	-	-
	F-M	0	-	-	-
	M-M	0	-	-	-
ICR	F-F	7	13.6 %	3.5 %	28.4 %
	M-M	0	-	-	-
	F-M	5	4.3 %	2.3 %	7.2 %
	M-M	1	0	0	0

The mean, minimum, maximum percentage of overlapping syllables in the all assigned syllables observed in each condition in each strain. The recording sessions with fewer than 10 assigned syllables from either mouse were excluded from this analysis. N, number of the sessions used.

Table S5. Detailed results of the two-way ANOVA in Fig. 2A, related to Figure 2.

		F(1,9)	*p*-value
**ICR, Female**	(vs F/M)	8.1058	0.0192*
	(R/I)	0.2727	0.6141
	(vs F/M) x (R/I)	10.0044	0.0115*
**ICR, Male**	(vs F/M)	5.1275	0.0498*
	(R/I)	0.0641	0.8059
	(vs F/M) x (R/I)	0.1553	0.7027
**B6, Female**	(vs F/M)	0.2791	0.6101
	(R/I)	0.5974	0.4594
	(vs F/M) x (R/I)	2.0187	0.1891
**B6, Male**	(vs F/M)	2.2182	0.1706
	(R/I)	0.0535	0.8223
	(vs F/M) x (R/I)	0.1290	0.7277

vs F/M, whether the partner was a female or a male; R/I, whether the subject was a resident or an intruder; $*p < 0.05$.

Table S6. Detailed results of the three-way ANOVA in Fig. 2B, related to Figure 2.

	df	F	*p*-value
(Action)	(7, 63)	12.815	4.1×10^{-10}***
(vs F/M)	(1, 9)	10.022	0.011*
(R/I)	(1, 9)	0.253	0.63
(Action) x (vs F/M)	(7, 63)	2.064	0.061
(Action) x (R/I)	(7, 63)	1.450	0.20
(vs F/M) x (R/I)	(1, 9)	9.705	0.012*
(Action) x (vs F/M) x (R/I)	(7, 63)	4.895	0.00019***

Action, the type of actions; vs F/M, whether the partner was a female or a male; R/I, whether the subject was a resident or an intruder; *$p < 0.05$; ***$p < 0.001$.

Table S7. Number of ultrasound segments that overlapped with BBVs, related to STAR Methods.

	Sub.	# of segments
B6	2<br>(F-F)	0
	2<br>(M-M)	1
	2<br>(F-M)	0
	2<br>(M-F)	3
	3<br>(M-FF)	0
ICR	2<br>(F-F)	99
	2<br>(M-M)	959
	2<br>(F-M)	834
	2<br>(M-F)	1999
	3<br>(M-FF)	1236

Sub., number of subjects in the cage; the first and second letters in parentheses indicate resident and intruder(s). M, one male; F, one female; FF, two females.
